# Construction and characterization of DNA libraries from cultured phages and environmental viromes

**DOI:** 10.1128/aem.01171-24

**Published:** 2024-09-24

**Authors:** Carmen Gu Liu, Brianna E. Thompson, James D. Chang, Lorna Min, Anthony W. Maresso

**Affiliations:** 1 Department of Molecular Virology and Microbiology, Baylor College of Medicine, Houston, Texas, USA; 2 TAILΦR LABS: Tailored Antibacterials and Innovative Laboratories for phage (Φ) Research, Baylor College of Medicine, Houston, Texas, USA; 3 Department of BioSciences, Rice University, Houston, Texas, USA; 4 Department of Medicine, Baylor College of Medicine, Houston, Texas, USA; Unversidad de los Andes, Bogotá, Colombia

**Keywords:** metagenomic libraries, DNA libraries, virome, bacteriophage, barcoded DNA, pooled DNA libraries, metagenomics

## Abstract

Despite many efforts to understand and leverage the functional potential of environmental viromes, most bacteriophage genes are largely uncharacterized. To explore novel biology from uncultivated microbes like phages, metagenomics has emerged as a powerful tool to directly mine new genes without the need to culture the diverse microbiota and the viruses within. When a pure computational approach cannot infer gene function, it may be necessary to create a DNA library from environmental genomic DNA, followed by the screening of that library for a particular function. However, these screens are often initiated without a metagenomic analysis of the completed DNA library being reported. Here, we describe the construction and characterization of DNA libraries from a single cultured phage (ΦT4), five cultured *Escherichia coli* phages, and three metagenomic viral sets built from freshwater, seawater, and wastewater samples. Through next-generation sequencing of five independent samplings of the libraries, we found a consistent number of recovered genes per replicate for each library, with many genes classifiable via the KEGG and Pharokka databases. By characterizing the size of the genes and inserts, we found that our libraries contain a median of one to two genes per contig with a median gene length of 303–381 bp for all libraries, reflective of the small genomes of viruses. The environmental libraries were genetically diverse compared to the single phage and multi-phage libraries. Additionally, we found reduced coverage of individual genomes when five phages were used as opposed to one. Taken together, this work provides a comprehensive analysis of the DNA libraries from phage genomes that can be used for metagenomic exploration and functional screens to infer and identify new biology.

**IMPORTANCE:**

Functional metagenomics is an approach that aims to characterize the putative biological function of genes in the microbial world. This includes an examination of the sequencing data collected from a pooled source of diverse microbes and inference of gene function by comparison to annotated and studied genes from public databases. At times, DNA libraries are made from these genes, and the library is screened for a specific function. Hits are validated using a combination of biological, computational, and structural analysis. Left unresolved is a detailed characterization of the library, both its diversity and content, for the purposes of imputing function entirely by computational means, a process that may yield findings that aid in designing useful screens to identify novel gene functions. In this study, we constructed libraries from cultured phages and uncultured viromes from the environment and characterized some important parameters, such as gene number, genes per contig, ratio of hypothetical to known proteins, total genomic coverage and recovery, and the effect of pooling genetic information from multiple sources, to provide a better understanding of the nature of these libraries. This work will aid the design and implementation of future screens of pooled DNA libraries to discover and isolate viral genes with novel biology across various biomes.

## INTRODUCTION

With the recent bloom of viral metagenomics through next-generation sequencing, the diversity of the global virome is being increasingly appreciated (1–3). Although viruses comprise the richest genetic diversity on Earth, more than 70% of their genome encodes proteins without known function, what we term the cryptic genosphere (4, 5). This gap between the number of discovered genes and an appreciation of their biological function has made understanding of the biology of this genosphere challenging. Hence, even though the computational assignment of function from sequenced metagenomes has accelerated our appreciation of the biology of environmental viromes, *in vitro* studies are still needed for the assignment and annotation of novel activities, especially when there is no similarity to the nucleotide and protein sequence, or a predicted structure, to anything reported in public databases.

Since the start of the metagenomics era, most studies have focused on characterizing sequence information to determine the types of species present in a sample set. Only a small number of studies focus on inferring function from *in vitro* screens (6). This is not surprising since the process is tedious and time-consuming. For example, it involves sampling from the environment (i.e., soil, hot spring, desert, or ocean), extracting the DNA, making DNA libraries by fragmenting the genetic material into clonable inserts, cloning into a desired vector, and expressing the environmental inserts in a host. Next, phenotypic screens are used to isolate genes relevant to the hypothesis of the study, and finally, the isolated genes are characterized (7, 8). This approach has been used to discover new classes of antimicrobials, antibiotic resistance elements, proteases, esterases, lipases, cellulases, pectinases, polymerases, nucleases, toxic resistance elements, transcriptional regulators, auxiliary metabolic genes, phage lysins, receptor binding proteins, Cas9 inhibitors, among many others (7–10).

Many of the challenges of this approach lie in the preparation of the DNA library. Important factors here include the preferred type and size of the library vector [plasmid, cosmid, fosmid or bacterial artificial chromosomes (BAC)], DNA fragmentation method (physical, chemical, or enzymatic), cloning strategy (homology-based or blunt ligation), vector specifications (antibiotics selection markers, inducers, and copy numbers), transduction or transformation (chemical or electroporation), and finally, the host for storage and expression (commercial or clinical bacteria, yeast, or eukaryotic strains; wild type or mutants) (11). Aside from considering the many available methods for DNA library construction, technical challenges during this phase include obtaining a high quality, high volume, and diverse source of DNA from the environment; identifying and removing contaminants, such as humic acids, that may interfere with downstream DNA amplification; low cloning and expression efficiency; and at times, the need to acquire costly equipment for DNA manipulation.

In recent years, many strategies have been developed to expedite the library building phase, including the expressible-linker amplified shotgun libraries (E-LASL) approach, which was adapted and developed by Schmitz et al. (12) for environmental viromes. Since viruses have small genomes, the total biomass recovered from uncultured environmental viromes is many logs in magnitude lower than the usual microbiomes, posing many challenges for functional metagenomics. Namely, there might not be enough starting materials for DNA construction, and if the purity of the DNA is low, there might not be enough for purification prior to construction. Hence, random amplification of the starting materials proved to be critical in these situations, and the E-LASL approach incorporates the amplification step within the construction phase (12).

However, despite the recently published strategies and gene discoveries, little is known about the specific composition of the DNA libraries that were created for most of the functional studies. Given the considerable amount of effort and time put into building DNA libraries as well as the many challenges that come with this process, it is critical that we understand the factors that may positively or negatively affect the final yield. By understanding the specific composition of the libraries constructed here, we may learn how to construct more diverse libraries, which may yield better opportunities to uncover new genes upon screening.

To work toward generating phage gene libraries, we used the previously developed E-LASL approach to construct DNA libraries from viruses isolated from freshwater, seawater, and wastewater samples. For comparison purposes, we also constructed DNA libraries from the *Escherichia coli* T4 phage as well as five different recently discovered *E. coli* phages. We pooled the clones together and assessed the pooling effect on repetitive sampling. We also investigated the consistency of each sampling by looking at the total genes and contigs, the median gene length, and the number of genes per contig harvested per replicate. We assessed the proportion of unknown proteins in each library and functionally classified them using KEGG and Pharokka. Finally, we mapped the sequencing data back to each phage genome or metagenomic crude extract to assess the overall coverage. Here, we report progress toward making phage gene libraries that can be used for functional metagenomic analysis and screening.

## RESULTS

### Construction and characterization of E-LASL from multiple metagenomic sources

**Fig 1:**
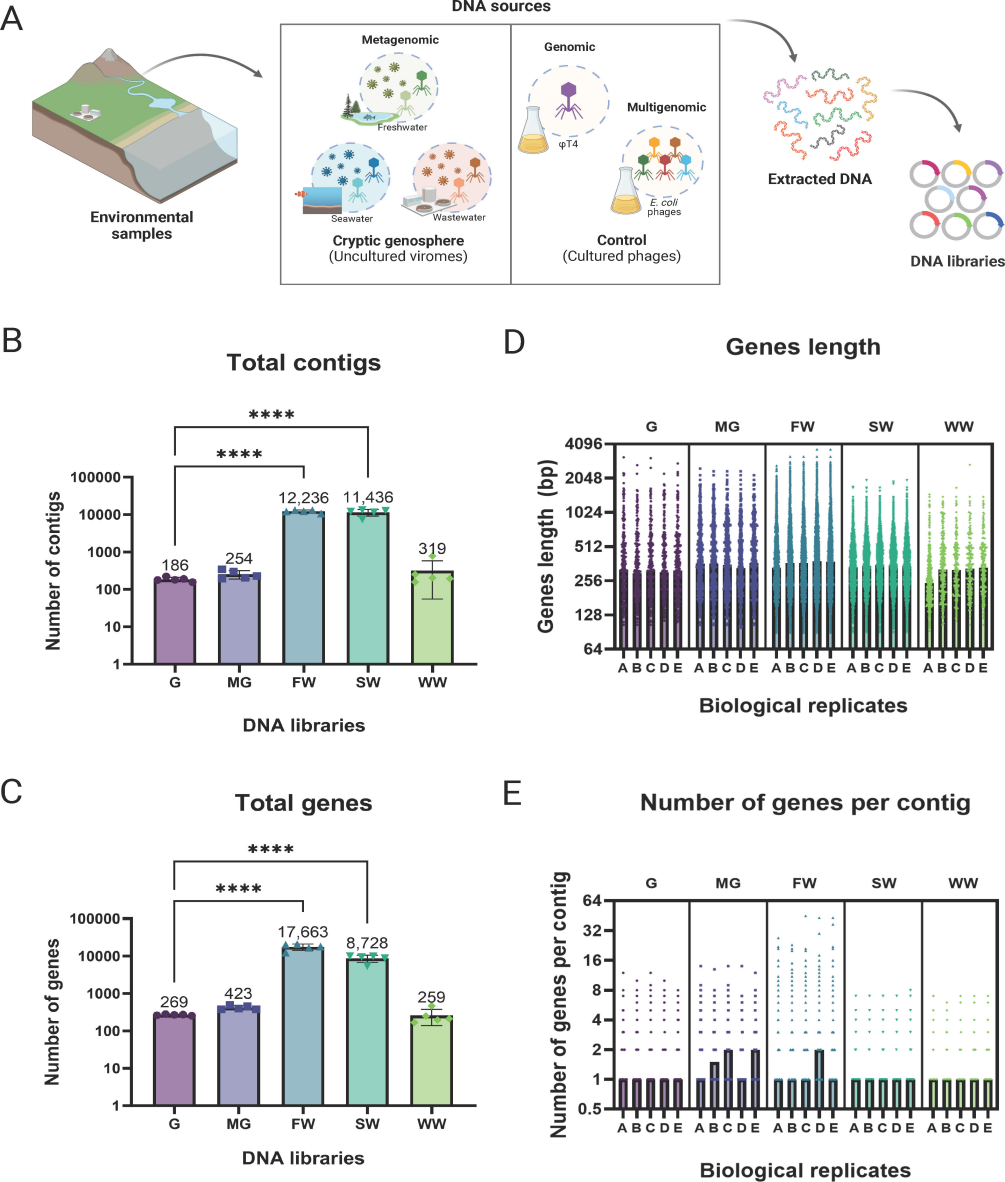
Construction and characterization of genomic, multigenomic, and metagenomic DNA libraries. (A) Freshwater (“FW”), seawater (“SW”), and wastewater (“WW”) viromes were sampled and processed for DNA library construction. As controls, T4 phage and five *E. coli* phages were included as genomic (“G”) and multigenomic (“MG”) libraries, respectively. After successful DNA library construction, plasmids were extracted, and amplicons were sent for sequencing. For each DNA library, the (B) total number of contigs per replicate, (C) total number of genes per replicate, (D) gene sizes, and (E) number of genes per contig are reported. For statistical significance, one-way ANOVA was performed. *P < 0.05; **P < 0.01; ***P < 0.001; and ****P < 0.0001. The median was graphed for the length and number of genes per contig. Each independent biological sampling of the DNA libraries is denoted as “A,” “B,” “C,” “D,” and “E” for a total of five replicates.

To construct DNA libraries from a rich source of genetic material, we sampled from various environmental sources totaling 360 L of water and enriched for their viromes via flocculation, centrifugation, and/or filtration (Fig. S1; Table S1). These viromes were grouped as metagenomic “freshwater,” “seawater,” and “wastewater” (Table 1; Table S1). Metagenomic samples were processed in a way that allows the retainment of mostly the viral portion with as little contaminants as possible. For instance, samples went through viral capture and three rounds of 0.22 µm filtration (Fig. S1). Prior to the DNA extraction, final samples were examined with transmission electron microscopy to ensure that no bacterial cells were present (Fig. S2), and extracellular nucleic acids that may contain bacterial origin were treated with DNase I as well as RNase prior to DNA extraction. As controls, we incorporated a “genomic” DNA library that consists of a single cultured phage (ΦT4) as well as a “multigenomic” library with five cultured *E. coli* phages (ФHP3, ФES17, ФHC8A, Ф6947, and Ф6948) (Table 1). Using the E-LASL approach as described previously, we inserted the extracted DNA into pBAD and constructed inducible DNA libraries (Fig. 1A). Together, we named these DNA libraries Inducible Multi MetagenФmic RecombinanT Libraries (IMMФRTL) to depict our main research goal of recovering genes capable of protecting and/or repairing bacterial genomes. The overall process involves (i) digesting the DNA with MlucI to smaller fragments and leaving overhangs, (ii) ligating the DNA fragments with adapters, (iii) amplifying the fragments with PCR to enhance cloning success, (iv) cloning the amplicons with overhangs to pBAD via TOPO-TA, (v) transforming the plasmids into *E. coli* BW25113 wild type, and (vi) pooling all transformants together per library (Fig. S3). Compared to the more intact genomic samples such as the DNA isolated from purified phages, our metagenomic samples have more fragmented DNA, which reduces the overall time needed for enzymatic digestion (Table 1).

**TABLE 1:** Characteristics of viral DNA libraries from single, multi-, and metagenomes

Libraries	Phage composition	Genome size (bp)	Digestion concentration and time	Total number of transformants obtained	Insert length (bp^*a*^)
Genomic	T4	168,903	0.2 U for 10 minutes	1,318	350–1,600
Multigenomic	HP3	168,188	0.2 U for 10 minutes	4,687	350–1,800
	ES17	75,134			
	6947	47,750			
	6948	44,545			
	HC8A	47,726			
	Total	383,343			
Metagenomic	Freshwater virome	N/A^*b*^	0.2 U for 5 minutes	90,180	350–2,500
	Seawater virome	N/A	0.2 U for 5 minutes	55,798	300–1,400
	Wastewater virome	N/A	0.2 U for 5 minutes	16,579	350–5,000

^*a*^ Estimated from agarose gel.

^*b*^ N/A, not applicable.

After transforming the libraries into *E. coli* BW25113, 10 random colonies were picked for confirmation (Fig. S3B). On average, 7 out of 10 colonies yielded insert sizes between 1,000 and 1,500 bp, while the rest had amplicons of ~200 bp, suggesting that 70% of the colonies contain enough insert sizes with coding potential (Fig. S3B). All transformants were pooled based on their library type: genomic, multigenomic, metagenomic freshwater, seawater, or wastewater. One of the advantages of pooling colonies includes efficient storage and distribution because large DNA libraries would often yield thousands of clones. As opposed to storing individual transformants in stacks of 96-well plates, thousands of colonies can be pooled, stored, and distributed in small vials. Most importantly, pooling clones together facilitates high-throughput screening and manipulation. For instance, the pooled library can be grown overnight and screened as a single culture instead of multiple 96-well plates for a single experiment.

The total number of transformants obtained ranged from 1,318 for the genomic library, 4,687 for the multigenomic, 90,180 for the freshwater, 55,798 for the seawater, and 16,579 for the wastewater (Table 1). While the number of transformants obtained for the metagenomic samples was arbitrary, the Clarke and Carbon formula allows the estimation of the number of clones required to cover the entire genome at least once with a certain threshold of probability (13). We used this formula to calculate the number of clones required to cover our genomic and multigenomic libraries with 99% probability. With this formula, we calculated 776 clones for the genomic library and 2,306 for the multigenomic library (Table 2). Since we recovered 1,318 clones for the genomic and 4,687 for the multigenomic, in essence, we recovered more transformants than required for both libraries (169%–203%). The total genome size for the metagenomic libraries is unavailable for the formula, yet through sequencing the crude extracts via shotgun metagenomics, we obtained the total number of base pairs per metagenome. We applied these data to estimate the number of clones needed to cover the metagenomes using the Clarke and Carbon formula and obtained 574,803 for freshwater, 627,321 for seawater, and 43,189 for wastewater (Table 2). In short, we obtained only 8.9%–38.4% of the estimated number of transformants required for the metagenomes; however, further analyses are needed to understand the actual coverage of the cloned genomes (Table 2).

**TABLE 2:** Estimating the total number of transformants required from sequencing crude extracts and functional libraries for complete coverage

Libraries	Genomic	Multigenomic	Metagenomic		
Phage composition	ΦT4	ΦHP3, ΦES17, Φ6947, Φ6948, ΦHC8A	Freshwater virome	Seawater virome	Wastewater virome
Genome size	168,903 bp^*a*^	383,343 bp^*a*^	124,817,309 bp^*b*^	136,221,491 bp^*b*^	9,378,825 bp^*b*^
Predicted number of transformants needed^*c*^	776	2,306	574,803	627,321	43,189
Total number of transformants obtained	1,318 (169% of prediction)	4,687 (203% of prediction)	90,180 (15.7% of prediction)	55,798 (8.9% of prediction)	16,579 (38.4% of prediction)
Actual coverage of functional DNA libraries	135,972 bp (80%)	279,832 bp (72.9%)	5,625,364 bp (4.5%)^*d*^	6,295,916 bp (4.6%)^*d*^	79,761 bp (0.85%)^*d*^
Estimated number of transformants to cover ~100% of the genomes/contigs	1,647	6,429	2,004,000	1,213,000	1,950,470

^*a*^ Total known genomic size.

^*b*^ Total sequenced nucleotides from the crude extract.

^*c*^ Based on the Clarke and Carbon formula.

^*d*^ Estimated via the covered contigs.

To confirm that DNA fragments were successfully cloned into vectors and transformed into *E. coli*, we extracted the libraries from frozen stocks, amplified the inserts, and analyzed them via electrophoresis (Fig. S4A). Overall, we saw a good proportion of amplicons of various sizes, suggestive of the diversity of the inserts (Fig. S4B). The range of the amplicons was similar among libraries: 350–1,600 bp for genomic, 350–1,800 bp for multigenomic, 350–2,500 bp for freshwater, 300–1,400 bp for seawater, and 350–5,000 for wastewater (Table 1; Fig. S4B). Note that the seawater library has a lower amplicon size range compared to the rest, even though the enzymatic conditions employed were the same as the other metagenomic libraries (Table 1; Fig. S4B). The genomic, multigenomic, and metagenomic wastewater libraries have distinct bands, suggesting bias of certain inserts in these libraries (Fig. S4B).

Although electrophoresis shows the presence and size of the inserts, they lack additional information relevant to the understanding of the libraries, such as the coverage, gene length, and functional classification. Therefore, we sequenced and analyzed five independent biological replicates of each library (Fig. S4A). Overall, *de novo* assembly produced contigs with a median length of 246–372 bp and a maximum length of 1,407–3,675 bp for all libraries (Fig. S4C). However, based on the gel electrophoresis data mentioned above, we believe that the contig lengths are an underestimate of the insert sizes, result of *in silico* assembly constraints of small amplicons. Strikingly, we found that resampling of the pooled DNA libraries produced a consistent total number of contigs and genes for each library, averaging 269 genes for genomic, 423 genes for multigenomic, 17,663 genes for freshwater, 8,728 genes for seawater, and 259 genes for wastewater (Fig. 1B and C). This means that each time the libraries were sampled for functional screens, hundreds to thousands of genes were potentially screened at a time. As a reference, the T4 phage genome contains 288 gene products for a total of 168,903 bp (accession no. NC000866) (14). By recovering ~269 genes for each sampling, this was the first indication that each replicate of the library does not cover the entire genome. Furthermore, note that metagenomic freshwater and seawater produced significantly more genes than the genomic library, averaging 65-fold more genes for freshwater and 32-fold for seawater. Even though this number of genes correlates to the high amount of transformants obtained for these libraries, we believe that genetic diversity plays a role here as well. For instance, we obtained 16,000 transformants for the wastewater library, which equals 12-fold more transformants than the genomic library. Yet, sequencing data show that we obtained only 1.7-fold more unique contigs for the wastewater library compared to the genomic library. We found that wastewater has less diversity compared to the other metagenomic samples due to the occurrence of a *Bacillus* phage that is highly prevalent in this library, representing 84% of the total reads from the crude extract (Tables S2 and S3).

Finally, we found that the genes had a median size of 303–322 bp for the genomic library, 330–366 bp for the multigenomic, 366–381 bp for the freshwater, 336–354 bp for the seawater, and 246–333 bp for the wastewater (Fig. 1D). For reference, the gene size for all phages found in both control libraries (ФT4, ФHP3, ФES17, ФHC8A, Ф6947, and Ф6948) was determined (Fig. S5A). In general, we found similar gene lengths when the control libraries were compared to the actual phages. For instance, phage T4 has a median gene size of 406 bp, while ФHP3, ФES17, ФHC8A, Ф6947, and Ф6948 have a combined median gene size of 352 bp (Fig. S5A). Moreover, to compare the gene size between our metagenomic libraries and other viromes, we randomly extracted 15 viral contigs from the IMG/VR v4 for each biome. In summary, we found the extracted viromes to have a median gene size of 438 bp for freshwater (*N* = 984), 438 bp for seawater (*N* = 525), and 393 bp for wastewater (*N* = 1,001), which are similar in size compared to our libraries (Fig. S5B). Finally, the median number of genes per contig was found to be between 1 and 2 for all libraries, with the highest number of genes per contig being freshwater (Fig. 1E).

### Metagenomic DNA libraries encode immense genetic potential suitable for diverse functional screens

Despite our efforts to minimize bacterial contamination, the presence of some level of bacterial DNA is unavoidable in virome studies as these can be present due to transduction or mobile genetic elements, as part of lysogenic cycles when phages incorporate adjacent bacterial genes into their genomes or could be present as misclassification by bioinformatic tools (15). To test for bacterial contaminants, ViromeQC was applied to each sample for all five libraries (15). On average, we find <5% alignment to both SSU rRNA (0.34%–1.4%) as well as the 31 prokaryotic single-copy markers (0.00021%–0.34%) for all libraries, indicating minimal non-viral contamination (Table S4). We then proceeded with the extraction of viral contigs through DeepVirFinder (16). On average, we extracted 59.4% contigs for the genomic library, 62.3% for multigenomic, 46.5% for freshwater, 18.01% for seawater, and 48.5% for wastewater (Fig. S6A). It was surprising that even though the genomic as well as the multigenomic library comprised 100% phages, only ~60% of the contigs were classified as “viral.” Since the vast majority of the viral reads are unknown, some of the viral contigs in these libraries could have been mistakenly classified as “non-viral,” leading to the underestimation of the viral portion. To find out the nature of these “non-viral contigs,” we mapped these reads back to their respective phages (ФT4 for the genomic library and ФHP3, ФES17, ФHC8A, Ф6947, and Ф6948 for the multigenomic), the vector pBAD, as well as the host *E. coli* MG1655 (Fig. S6B). In summary, we found almost half of the “non-viral contigs” of the control libraries to be viral (56% for genomic and 48% for multigenomic), with the rest mapped to pBAD (8.5% for genomic and 12.3% for multigenomic), the host *E. coli* (21.6% for genomic and 13.3% for multigenomic) as well as unmapped (13.5% for genomic and 26% for multigenomic) (Fig. S6B). The reads that mapped back to the host were briefly examined in the genomic library, and we found that most of these reads were either directly on or adjacent to “mobile genetic elements” and “prophages” found in the ФT4 genome (data not shown), suggesting that some gene shuffling may have occurred during phage infection. In summary, the data lead us to believe that some “non-viral” contigs were actually viral, and presumably, this is the case for freshwater, seawater, and wastewater as well.

**Fig 2:**
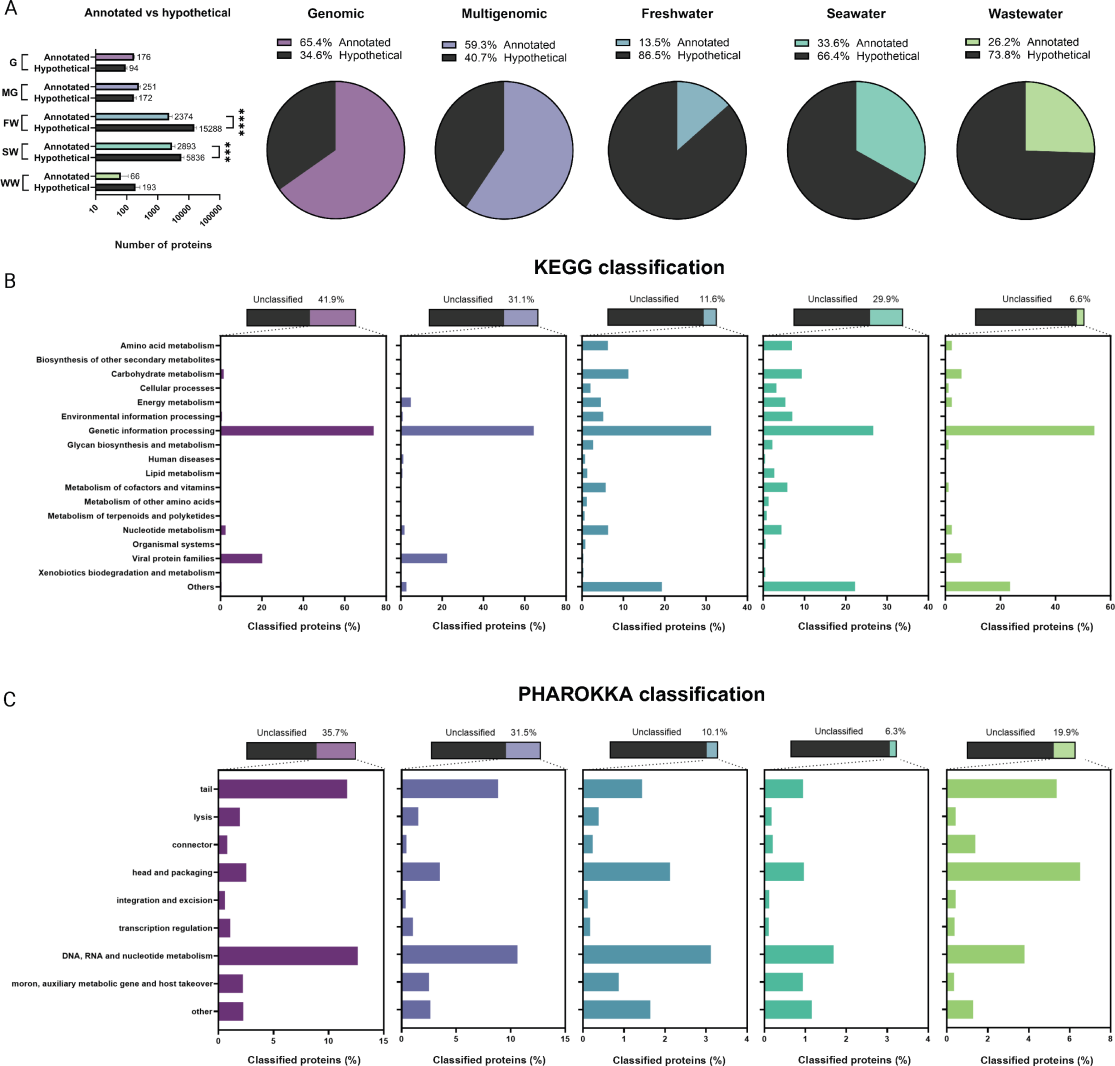
Annotation and classification of proteins recovered from genomic, multigenomic, and metagenomic DNA libraries. (**A**) The number of annotated and hypothetical proteins for each DNA library: genomic (“G”), multigenomic (“MG”), freshwater (“FW”), seawater (“SW”), and wastewater (“WW”) classified by Prokka and Rast. Pie graphs show the average percentage of annotated vs hypothetical proteins from five biological replicates. (**B**) Amino acid sequences from each DNA library were classified in the KEGG system for their potential roles in different biological pathways. (**C**) All libraries were also classified with Pharokka for viral genes. For statistical significance, a two-way ANOVA was performed. *P < 0.05; **P < 0.01; ***P < 0.001; and ****P < 0.0001.

To further characterize the genetic potential of each library, all coding sequences were annotated according to their functional roles. On average, each sampling of the genomic library produced 176 counts of annotated proteins that make up 65.4% of the total and 94 hypothetical sequences that make up 34.6% of the total (Fig. 2A). As a reference, 107 out of the 288 gene products in the T4 phage genome are currently annotated as hypothetical, which constitutes 37% of the genome (accession no. NC000866) (14). This indicates that the percentage of the annotated coding sequences in our genomic library is mostly consistent with what is currently known with the T4 phage genome, an indication of the absence of genome-wide coverage bias. Similar to the genomic library, the multigenomic library contains more annotated than hypothetical coding sequences. On average, each sampling yielded 59.3% (*N* = 251) annotated and 40.7% (*N* = 172) hypothetical sequences (Fig. 2A). It is reasonable that the multigenomic library has a slightly lower proportion of annotated sequences since these phages are not as widely studied and characterized as ΦT4, resulting in more potential for unknown proteins. However, it was surprising that only 40% of the sequences were uncharacterized, considering that the multigenomic library contains five different sources of phage DNA recently discovered in the laboratory.

Conversely, the metagenomic libraries possess a larger percentage of hypothetical sequences. In particular, each sampling of the freshwater library resulted in only 13.5% of annotated (*N* = 2,374), while the rest of 86.5% were hypothetical sequences (*N* = 15,288, Fig. 2A). The seawater library contains 33.6% annotated (*N* = 2,893) and 66.4% hypothetical sequences (*N* = 5,836, Fig. 2A). Finally, the wastewater library resulted in 26.2% annotated (*N* = 66) and 73.8% hypothetical sequences (*N* = 193, Fig. 2A). In short, hypothetical proteins make up 66.4%–86.5% of our metagenomic DNA libraries. It is noteworthy that although both genomic and wastewater libraries produced roughly the same number of genes per sampling (Fig. 1D), the majority of these genes (73.8%) in the wastewater library are unknown, while only 34.6% of the genomic library is unknown. This further confirms the value of metagenomic sources of DNA, which has more potential of possessing novel genes than individual phage genomes, albeit the roughly same number of genes were cloned into these libraries.

Then, we attempted to classify the recovered gene products into various biological pathways for comparison purposes. Regardless of their annotations, the coding sequences in each library were classified based on the KEGG system into various defined biological pathways. Despite the recent incorporation of the new viral data set named virus ortholog clusters into KEGG, classification of viral proteins remains a challenge. As a result, only a small number of proteins were classified in this system (Fig. 2B). For instance, only 6.6%–41.9% of the total proteins in the libraries were classified (Fig. 2B). Nonetheless, most classified proteins (>60%) in both genomic and multigenomic libraries were found to be involved in “genetic information processing” or as “viral proteins” (Fig. 2B). In comparison, all metagenomic libraries have proteins classified into multiple biological pathways in addition to genetic information processing (Fig. 2B). These include amino acid metabolism, nucleotide metabolism, carbohydrate metabolism, cellular processes, energy metabolism, environmental information processing, glycan biosynthesis and metabolism, lipid metabolism, and metabolism of cofactors, vitamins, and other amino acids (Fig. 2B). Again, even though only 6.6% of the proteins were classified in the wastewater library compared to the 41.9% in the genomic library and that both have similar number of genes per sampling (Fig. 1D), metagenomic wastewater contains proteins involved in a diverse set of pathways (Fig. 2B). In summary, not only do metagenomic libraries contain more unknown genes for novel discoveries but also the small subset of classified proteins further corroborates the notion that metagenomic DNA libraries encode immense potential for genetic diversity involved in multiple biological pathways.

Finally, we classified the contigs with PHAROKKA, which is a tool designed specifically for phage genomes (17). In agreement with the previous KEGG classification, PHAROKKA also detected a substantial number of genes involved in DNA, RNA, and nucleotide metabolism for all libraries (Fig. 2C). The next most abundant classified genes fall into the category of “tail” as well as “head and packaging” for all libraries. Note that the KEGG classification failed to detect “viral protein families” for the metagenomic libraries, but with PHAROKKA, genes involved in tail, lysis, connector, head, and packaging were detected in the metagenomic libraries (Fig. 2B and C).

### DNA library cloning coverage differs from the predicted number of transformants

**Fig 3:**
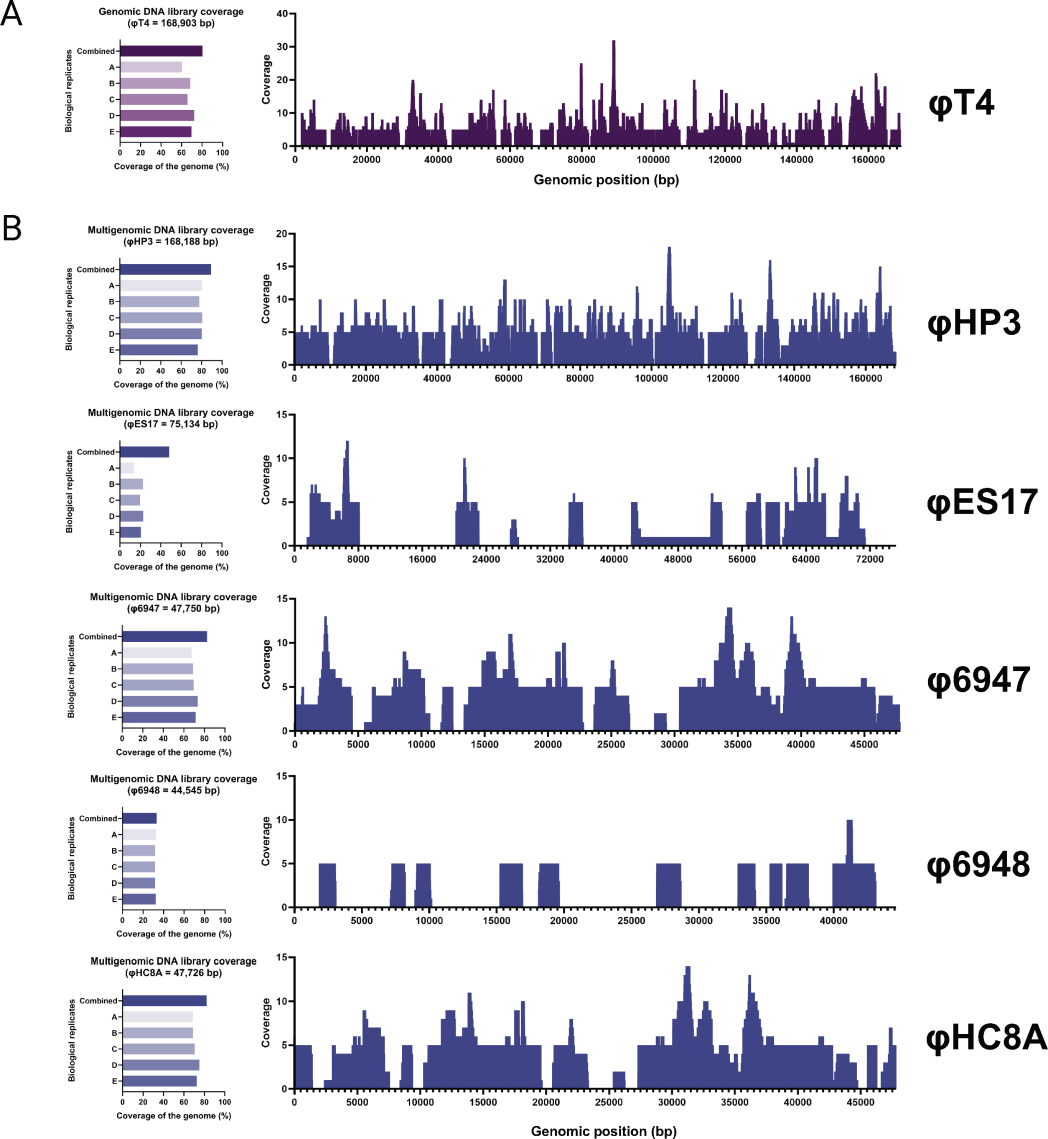
Coverage maps for genomic and multigenomic DNA libraries. Assembled contigs recovered from each individual DNA library replicate were mapped to their reference genomes. In addition, all five biological replicates were combined and mapped to their respective genomes. (**A**) Genomic DNA library coverage by ΦT4. (**B**) Multigenomic DNA library coverage by ΦHP3, ΦES17, Φ6947, Φ6948, and ΦHC8A. Coverage maps were shown with the combined replicates. Each independent biological sampling of the DNA libraries is denoted as “A,” “B,” “C,” “D,” and “E” for a total of five replicates.

Although theoretically we obtained enough clones to cover the entire phage genome for both our genomic and multigenomic libraries based on the Clarke and Carbon formula, we proceeded with mapping our sequencing data back to the reference genomes to understand the actual coverage of each library. As a result, we observed that each time we sampled from the genomic library, we essentially harvested DNA that covered 60%–70% of the ΦT4 genome (Fig. 3A). When all five replicates were combined, the coverage of ΦT4 reached 80.5% (Fig. 3A). As stated above, we obtained *N* = 1,318 transformants in contrast to the predicted *N* = 776, which is 169% of what was presumably required (Table 2). However, the DNA library data show less cloning coverage, which indicates that future DNA library construction should aim above the estimated number of transformants for a more complete coverage. Interestingly, genome-wide analysis showed that coverage was even throughout, and it did not seem that there was a region that was biasedly cloned (Fig. 3A).

**Fig 4:**
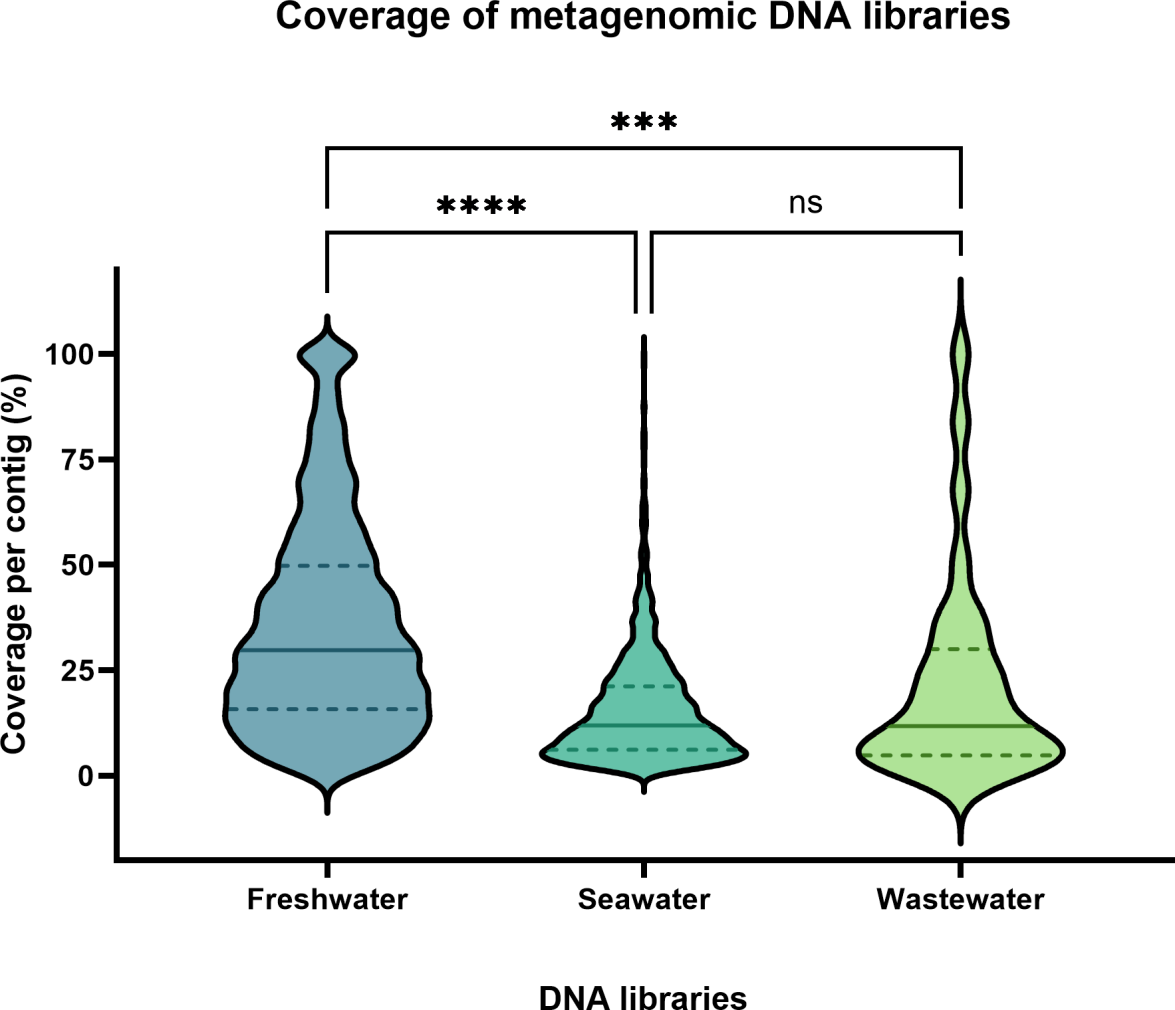
Coverage for metagenomic DNA libraries. Assembled contigs from all five biological replicates were combined and mapped to their metagenomic contigs. Kruskal-Wallis test was performed for statistical comparison. *P < 0.05; **P < 0.01; ***P < 0.001; and ****P < 0.0001.

As a whole, the multigenomic library covered 72.9% of all phage genomes. Yet, based on the number of transformants obtained vs predicted, we obtained 203% more transformants than the predicted number obtained from the formula of Clarke and Carbon (Table 2; Fig. 3B). We also noticed that when five different phage genomes were used for cloning, certain phages had reduced coverage. Namely, we obtained 89.3% combined coverage for ΦHP3, 48.1% coverage for ΦES17, 82.6% coverage for Φ6947, 33.2% coverage for Φ6948, and 82.2% coverage for ΦHC8A (Fig. 3B). Interestingly, it did not seem that larger genomic fragments of the phage positively correlate to the higher success of cloning in our case. For instance, ΦES17’s genome is larger (75 kb) compared to that of Φ6947 (47 kb), but ΦES17 was less covered than the latter (Fig. 3B). Since we restricted the initial input of each phage DNA to be 100 ng for a total combined 500 ng for DNA library construction, we do not believe that the abundance of the phage may have contributed to this large variation as there would be more genomes of the smaller size phages leading to higher chances of being cloned. Yet, smaller genomes were not more covered than the larger genomes in this study. Differential cloning is also evident with metagenomic libraries, as we mapped the sequencing data of each cloned library back to each assembled contig obtained from shotgun sequencing the crude extracts. We found that coverage of all contigs in the freshwater DNA library ranged from 0.2% to 100%, with a median coverage of 29.8% (Fig. 4). The median coverage for seawater and wastewater libraries was even lower, estimated at 11.9% and 11.8%, respectively (Fig. 4).

We assessed the expression of the vector and found that under uninduced conditions, there was negligible expression of the recombinant proteins (data not shown), suggesting that potential leakiness of the promoter is unlikely the cause of the uneven coverage of the phage genes. This is corroborated by other studies in which they have also found negligible expression under uninduced conditions with the pBAD promoter (18–20). By assessing the presence and coverage of known toxic genes, we may understand whether toxic genes play a role here. Based on previous studies, the endolysin (gene *e*), holin (gene *t*), nucleoid disruption protein (gene *ndd*), and endoribonuclease (gene *regB*) from the phage T4 have been shown to cause toxicity in *E. coli* (21–23). When we assessed the mapping of the genomic library, we found that all the abovementioned genes were covered, suggesting that toxic genes may not be the cause of the differences in coverage (Table S5). As for the multigenomic control, we found the presence of holins, lysozymes, and/or lysins in the cultured phage genomes; however, the toxicity of these enzymes from our cultured phages has not been directly tested yet. We found in total eight of these products in the phage genomes and four out of eight were covered by the library found in three out of five phages (Table S5). It is not clear whether the potential toxicity of such genes was the cause of cloning differences in these phages for the multigenomic library. However, since half of them were covered in our multigenomic library and the known toxic genes were covered in the genomic library for the phage T4, toxic genes, in general, are unlikely the cause of the differential coverage of the libraries in our case. Yet, other possibilities may include regions that are difficult to clone, such as secondary DNA structures (i.e., hairpins, repetitive sequences), which could also be contributing factors.

To understand the success of our metagenomic libraries compared to what is available from our crude extracts, we divided the total number of nucleotides obtained from mapping the DNA libraries to the contigs over the total number of nucleotides obtained from sequencing the crude extracts (Table 2). In short, we obtained 4.5%, 4.6%, and 0.85% total coverage for freshwater, seawater, and wastewater, respectively. In other words, there was a large fraction of DNA amounting to 95%–99.1% of the total genetic material that was left out from our DNA libraries, despite the diversity of our existing genes as shown above. We then used these data to estimate the number of clones needed for complete coverage and obtained at least 1.2–2 million clones per metagenomic library (Table 2). These estimates further confirm that the Clarke and Carbon formula is not applicable for metagenomic samples, as the total number of nucleotides is difficult to estimate. Altogether, our data suggest that sequencing the cloned libraries is a better approach to understanding the actual coverage of the libraries. This proves to be useful during the construction phase, and we recommend obtaining as many clones as feasible for the metagenomes, as the genetic diversity is vastly unmeasurable for such libraries.

### Effects of pooling DNA libraries for better storage and high throughput functional screens

**Fig 5:**
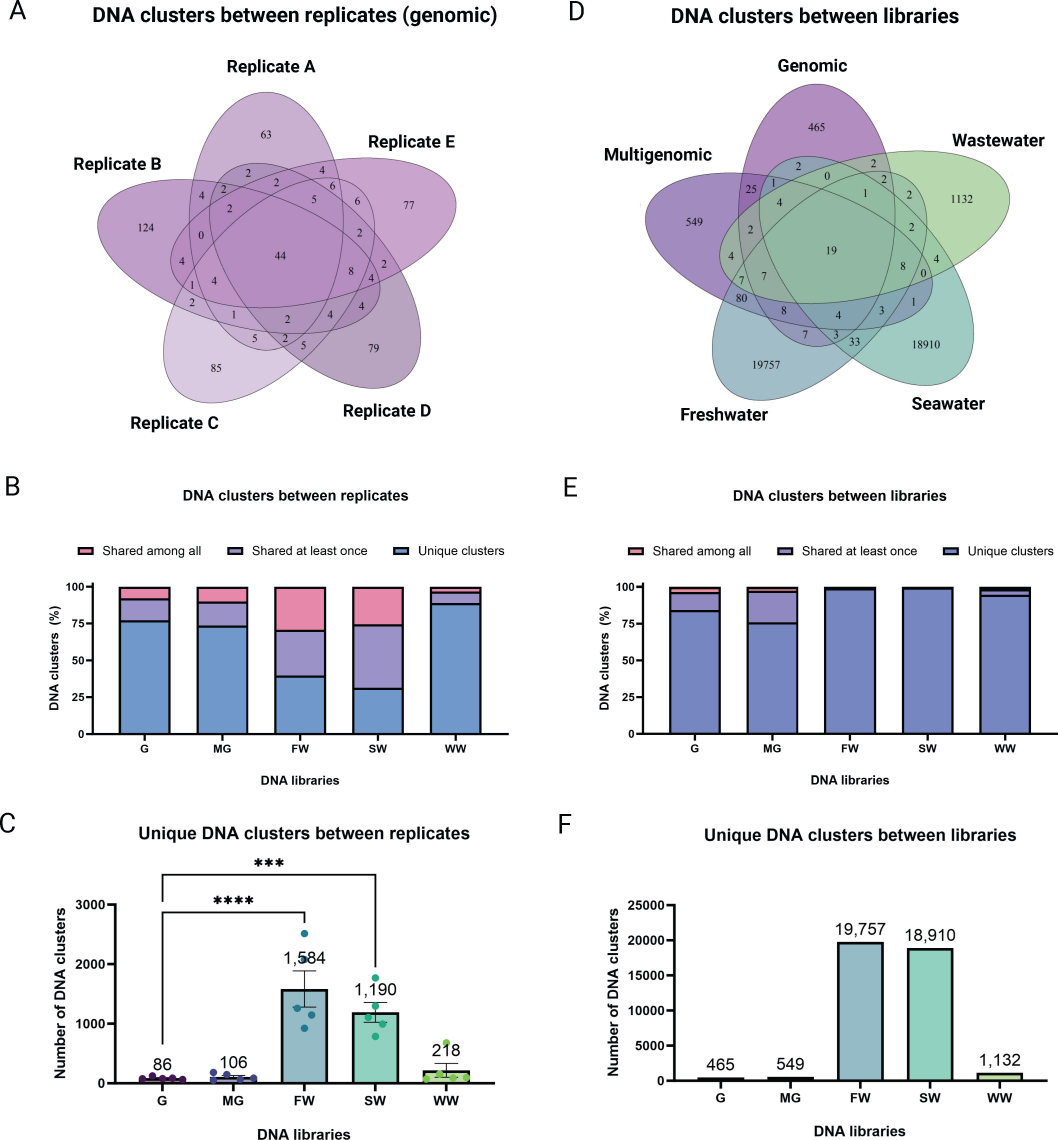
Unique and shared DNA sequences among replicates and libraries. DNA sequences from each library were clustered between replicates and represented as (A) a Venn diagram showing the exact counts of unique and shared DNA clusters between replicates; (B) the percentage of DNA clusters that are unique and shared among all replicates; and (C) the total number of unique DNA clusters that are not shared between replicates. In addition, sequences from all libraries were clustered and represented as (D) a Venn diagram showing exact counts of unique and shared DNA clusters between libraries; (E) the percentage of DNA clusters that are unique and shared among libraries; and (F) the total number of unique DNA clusters that are not shared among libraries. For statistical significance, one-way ANOVA was performed. *P < 0.05; **P < 0.01; ***P < 0.001; and ****P < 0.0001. Each independent biological sampling of the DNA libraries is denoted as “A,” “B,” “C,” “D,” and “E” for a total of five replicates.

In this study, DNA libraries were created and pooled based on the sample type. Given the diversity of our sources, it was assumed that different genes may be harvested each time the libraries were sampled from the frozen stocks. Yet, the percentage of the same vs different sequences for each sampling was unknown. Hence, we clustered DNA sequences between different biological replicates based on 95% identities for each library (Fig. 5A; Fig. S7). As a result, we found that there are DNA clusters unique for each independent sampling, sequences that are shared at least once with other replicates, and sequences that are shared among all replicates (Fig. 5A; Fig. S7). By computing the percentage of the DNA clusters in these categories, we found that genomic, multigenomic, and wastewater have 77%, 73%, and 88% DNA clusters that are unique for each independent sampling, respectively (Fig. 5B). In contrast, freshwater and seawater only produced 39% and 31% unique sequences per replicate, respectively (Fig. 5B). In addition, for genomic, multigenomic, and wastewater libraries, we only recovered <10% of sequences that are shared among all five biological replicates (Fig. 5B). While up to 29% and 25% of identical sequences shared among all five replicates were recovered from freshwater and seawater libraries, respectively (Fig. 5B). In summary, these data indicate that there are more chances of recovering highly identical sequences per replicate for the freshwater and seawater libraries, and more unique genes for genomic, multigenomic, and wastewater libraries. Yet, despite the higher chances of screening the same sequences in freshwater and seawater libraries, by sheer volume, these libraries produce more unique DNA sequences per replicate when compared to the genomic library (Fig. 5C). Namely, each time we sample from the genomic library, we are essentially recovering only 86 unique clusters of sequences as opposed to 1,584 (18-fold) and 1,190 (14-fold) from freshwater and seawater, respectively (Fig. 5C).

To understand the genetic similarity between DNA libraries, we also clustered sequences between libraries. As a result, Fig. 5D shows that only 19 DNA clusters are shared among all libraries with the rest of them being unique DNA sequences per library. For example, the genomic library resulted in 465 unique clusters (84.2%), multigenomic in 549 clusters (76.0%), freshwater in 19,757 clusters (99.1%), seawater in 18,910 clusters (99.5%), and wastewater in 1,132 clusters (94.6%) (Fig. 5D through F). Altogether, these data show the uniqueness of each library. Even though genomic and multigenomic are less diverse, these libraries have their intrinsic value for novel gene discovery, as their sequences are not shared in the metagenomic libraries.

## DISCUSSION

In summary, we present our comprehensive report on the functional metagenomic libraries that were generated from uncultured environmental viromes as well as their control genomic and multigenomic libraries generated from cultured phage genomes. As part of our functional studies, we sequenced the libraries to understand essentially what genes were being screened each time the library stocks were sampled. This study can serve as a proof-of-concept on how pooled DNA libraries can be rapidly sequenced and analyzed for quality, number of genes, potential for unknown, differences in sampling, and coverage for bacterial viruses from terrestrial sources. The primary highlights of this study can be summarized as follows: (i) our DNA libraries contain contigs with a median length of 246–372 bp and maximum length of 1,407–3,675 bp for all libraries with an average of one to two genes per contig; (ii) the amount of genes recovered per library is strikingly consistent as represented in five independent biological replicates; (iii) the median gene size is 303–381 bp, suitable for viral metagenomics; (iv) the majority of the unknown sequences and major diversity in functional potential are derived from metagenomic samples, even though the same amount of genes were recovered in two libraries (i.e., genomic vs wastewater); (v) increasing sample diversity, even with only five small phages in our case, introduces bias cloning due to randomness or difficult-to-clone regions not examined here (i.e., multigenomic and metagenomic); (vi) pooling DNA libraries does produce unique DNA clusters per sampling; however, this is library dependent (i.e., genomic vs freshwater and seawater); and finally, (vii) the DNA libraries constructed here do not share highly similar DNA sequences, even with the genomic and multigenomic libraries. In a sense, each library has its own valuable genetic source for the contribution of novel gene mining.

In addition to the highlights mentioned above, many lessons were learned from this study for pooled libraries. First, based on the highly consistent sampling data obtained from five independent biological replicates, we found that only one sequencing attempt per library would be sufficient to estimate the number of genes, gene size, genes per contig, number of hypothetical vs annotated sequences, and overall coverage per library. All these could serve as indicators of the quality of the library. Clearly, the more independent replicates, the better, but estimating the overall coverage of the library during the production phase with only one sampling would allow us to estimate how many more clones are needed for more coverage with minimum resources. That is, it would allow us to estimate whether to continue building the libraries or proceed with phenotypic or selection screens. Second, we found that pooling transformants in a library does produce a substantial number of unique DNA sequences per sampling. Although the proportion of the unique sequences per sampling seemed to be lower in the most diverse libraries (i.e., freshwater and seawater), there will still be thousands of new genes to be screened in functional assays by the sheer volume and diversity of the libraries. Note that the expression of these genes depends heavily on the compatibility with the host in terms of codon usage, codon codes, potential toxins, and many other factors (24). The current characterization of the libraries is with the cloned genes or DNA fragments and we hope to report on these studies in future manuscripts. Third, we recommend increasing the number of transformants as much as possible for metagenomic libraries to get higher coverage. Even with thousands of transformants per library, we were only able to clone a fraction of what was readily available from our crude extracts. Using the total number of nucleotides from sequencing data as a guide, we can estimate the progress of constructing the libraries as well. Finally, we suggest increasing the volume of environmental sources for library construction, especially for viromes. A challenge that could be encountered is the amount of starting DNA needed for optimization. For example, the initial digestion step required five times the starting materials to determine the time required (undigested, 3, 5, 10, and 15 minutes) for any particular DNA sample. These were sacrificed for gel electrophoresis, and a new round of digestion was needed for the actual library construction. Given that each digestion was done with 500 ng of DNA, eventually, we used at least 3 µg of DNA per library. As a reference, even in biomass-rich stool samples, 1 g of a stool sample yields between 0.22 and 0.87 ng/µL of phage DNA (25). If we consider 50–200 µL as elution volume, then 1 g of stool may only yield between 11 and 174 ng of DNA as opposed to 3 µg needed. In our study, we harvested 100–200 L for freshwater and seawater since the usual volume reported in other studies fall in the range of 20–100 L (26, 27). Whereas for wastewater we harvested 16 L as this sample is highly dense in microbiomes and viromes. Studies have reported as low as 1 mL to 1 L of wastewater samples for metagenome analysis (28, 29). Hence, processing large amounts of environmental samples and obtaining large DNA volumes are highly recommended for library construction, especially for environmental viromes. This also highlights the need for the use of alternative methods and/or technologies to enhance viral capture from large volumes or a significant mass of environmental samples.

Characterizing metagenomic libraries is rarely reported. A previous study reported on the characterization of Archaeal metagenomes, in which three large-insert fosmid libraries were created from soil samples, but their focus was on characterizing the relatedness to the actual microbiome and not on the success of the library (30). Another study reported on the construction of BAC libraries from a single genome, *Xenopus tropicalis*, and characterized the coverage of the library. Yet, this study only had one library built, and so there was no comparison made with other libraries in the same study. In addition, both studies individually sequenced hundreds to thousands of clones, resulting in a time-consuming and costly process, and thus not comparable to the present study. Here, we present our characterization of pooled, small-insert, viral DNA libraries from various sources.

Currently, these libraries are being used in our laboratory for the discovery of novel genes involved in defending cells against reactive oxygen species, novel RNA polymerases, and novel DNA repair enzymes. However, other cell functions can be interrogated as well, including screens related to metabolism or protein synthesis and so forth. Furthermore, there are several additional types of approaches that can be performed with pooled libraries. The first method is a phenotype screen, in which a pooled library is diluted either on agar plates or on 96-well plates and screened for any phenotype of interest (31). For instance, the presence of phage lysins or antimicrobial resistance elements. Another method could be a positive selection screen, where the selective pressure allows only the survival of selective clones from a large population. In this strategy, DNA libraries can be expressed in mutants for heterologous complementation and an external stressor could be added as another selective pressure (31). Examples include finding novel catalases, polymerases, and nucleases by introducing these libraries in mutants with single-gene deletions (i.e., KEIO collection). Other screens involve reporter systems like the *gfp* gene and could be used to discover aromatic-hydrocarbon-induced genes, small molecules, and amidases (31).

Future improvements on metagenomic libraries used for functional screens may include building a centralized system in which DNA libraries are reported and shared. Sourcing from diverse samples of environmental microbes is within our immediate interests. DNA libraries can be expanded and varied by organisms (viral, bacterial, or fungal), sources (desert, forest, soil, water, leaves, or sand), and vectors (plasmids, cosmids, or BAC). Since our initial interests were in viral genes, which are usually shorter and more compact, the vector of choice used here was suitable. However, cosmids or BACs should be considered for larger inserts such as bacterial or fungal genomes. Other improvements could involve expanding the expression of the libraries since there are various factors and criteria in place that make a DNA library expressible, such as the average gene size based on the biome, promoters, and transcription machineries in the host, among many others. Some solutions may include engineering ribosomes to improve *E. coli* as an expression host or making the libraries themselves suitable for many hosts with expanded transcription machinery.

To summarize, here we report the characteristics of our pooled genomic, multigenomic, and three metagenomic DNA libraries through next-generation sequencing. Using this method, we tested five independent replicates of each library and analyzed their gene content, coverage, and effects of pooling and sampling. We observed that each sampling is consistent in reporting the overall depth of the libraries, which can be used as a quick measure during the library building process to estimate the amount of transformants needed for higher coverage. We also found that although roughly the same number of genes are recovered per sampling, the gene content differs. This ensures that each time we sample from a pooled library, we are harvesting unique genes for the functional screens. Overall, our characterization of the DNA libraries sheds light on how future DNA libraries can be improved based on the factors discussed in this study. We believe libraries sourced from microbial biomes are critical to assigning function to uncharacterized cryptic genes and expanding knowledge of life’s biological possibility.

## MATERIALS AND METHODS

### Bacterial and phage storage, growth conditions, and purification

*E. coli* BW25113 was obtained as part of the KEIO collection (Table S6). Bacterial cultures were stored in −80°C with 25% glycerol. Phages were obtained from ATCC and TAILΦR Labs and stored in glass vials at 4°C (Table S6). Phages were amplified by first making a plate stock with the bacterial host, a small batch culture (25 mL), and then a large batch culture (4 L) as described previously (32). To recover phages, NaCl (30 g/L) and PEG8000 (7.5%) were added for overnight precipitation, and the pellet was harvested by centrifugation at 10,000 rpm for 50 minutes at 4°C. Clean-up was performed by adding chloroform (1:1, vol/vol), centrifuging at 10,000 rpm for 10 minutes, collecting the aqueous phase, and adding DNase and RNase (final 5 µL/mL). Phages were purified by isopycnic CsCl gradient centrifugation (30,000 rpm, 10°C, 18 hours), and phage bands were harvested and dialyzed in phage buffer (1:1,000).

### Bacteriophage isolation from environmental samples

#### Freshwater

Approximately 220 L of freshwater was collected from creeks, lakes, wetlands, and rivers around Austin and Houston, TX, USA (Table S1). These samples were combined for further processing. To remove large particles, freshwater was first filtered with Whatman GF/A borosilicate fine retention prefilter (1.6 µm pore size, catalog no. 28497-255) (Fig. S1). To remove bacterial contaminants, filtered water was processed with Millipore Express Plus hydrophilic polyethersulfone (PES) filter (0.22 µm pore size, catalog no. GPWP14250, EMD Millipore). The viral fraction (flow-through) was flocculated with FeCl_3_ for at least 1 hour (final concentration of 1 mg/L of Fe) and collected with Millipore Isopore hydrophilic polycarbonate membrane filter (0.8 µm pore size, catalog no. ATTP14250, EMD Millipore) (33). The flocculants were resuspended overnight at 4°C in the dark with 1 M citrate magnesium buffer (1 M citrate dihydrate, 0.05 M magnesium chloride hexahydrate, pH 6.5, 1 mL of buffer per 1 L of water) (34). The resuspension was transferred to a fresh tube, and the old filters were centrifuged at 500 × *g*, 4°C for 3 minutes to collect the remaining fluid. To remove potential bacterial contaminants, the water sample was filtered again with PES. To further concentrate the water samples, Amicon 100 kDa MWCO ultrafiltration was performed with 3,000 × *g* at 4°C. After sample recovery, 1.5 mL of phage buffer (100 mM NaCl, 6.7 mM Tris-HCl, 3.2 mM Tris-Base, and 10 mM MgSO_4_, pH 8.0) was added to the upper reservoir and vortexed briefly to further recover bound phages on the filter. This was combined with the recovered fraction to constitute the final sample.

#### Seawater

Approximately 110 L of seawater was collected from the coast around Houston, TX, USA (Table S1) and processed as described above for the freshwater sample using chemical flocculation with FeCl_3_ for viral recovery (Fig. S1).

#### Wastewater

Approximately 16 L of wastewater was collected from several wastewater treatment plants around Austin and Houston, TX, USA (Table S1). To disrupt the hydrophobic interactions between viruses and solid particles, glycine (0.25 M, pH 9.5, 1:3, vol/vol) was added to the samples and stirred at 4°C for at least 2 hours. To remove bacterial contaminants, filtered water was processed with a Millipore Express Plus hydrophilic polyethersulfone filter (0.22 µm pore size). To concentrate the viral fraction, 2.5 M MgCl_2_ was added to the sample (1:100 dilution) for a final 25 mM of MgCl_2_, and the sample was filtered via HA filters (mixed cellulose ester, 0.45 µm pore size, catalog no. HAWP04700, EMD Millipore). Filters were collected into a falcon tube, and 5 mL of 1 mM NaOH was added to each filter. This solution was neutralized with H_2_SO_4_ (final concentration 0.5 mM). To remove potential contaminants, the sample was filtered with PES (0.22 µm pore size). Final wastewater samples were concentrated with Amicon 100 kDa MWCO ultrafilter (3,000 × *g*, 4°C). To further concentrate the wastewater, Amicon 100 kDa MWCO ultrafiltration was performed with 3,000 × *g* at 4°C. After sample recovery, 1.5 mL of phage buffer (100 mM NaCl, 6.7 mM Tris-HCl, 3.2 mM Tris-Base, and 10 mM MgSO_4_, pH 8.0) was added to the upper reservoir and vortexed briefly to further recover bound phages on the filter. This was combined with the recovered fraction to constitute the final sample.

#### Transmission electron microscopy

Each sample (2 or 5 µL) was placed on a formvar/carbon film nickel grid, 200 mesh (Electron Microscopy Sciences, catalog no. FCF200-Ni) that had been treated for 30 s with a PELCO Easy Flow glow discharge instrument to increase the hydrophilicity of the grid. After the material settled and adhered to the grid for 1 minute, the liquid droplet was removed with blotting paper, and the grid was washed with 5 µL of the negative stain, 1% uranyl acetate. This stain solution was immediately removed with blotting paper and a second 5 µL of stain was added and allowed to remain on the sample for 30 s. The grid was blotted dry and then dried in air. The grids were imaged on a JEM1400 transmission electron microscope (JEOL) operated at 120 kV.

### Extraction and purification of DNA from cultured phages and environmental samples

#### Cultured phages

After CsCl purification, phage DNA was extracted via the EZNA Universal Pathogenic Kit (catalog no. D4035-01, Omega Bio-tek) according to the manufacturer’s instructions. DNA was eluted with 70°C nuclease-free water.

#### Metagenomic samples

Before all DNA extractions, the final concentrated samples were filtered once again with PES 0.22 µm filters to remove potential bacterial contamination. To remove free nucleotides, DNase I (final 20 µg/mL) and RNase (final 40 µg/mL) were added, and the solution was incubated at 37°C for 1 hour and heat-inactivated at 75°C for 10 minutes. Then, DNA was extracted via phenol chloroform isoamyl alcohol (PCI). Briefly, samples were digested by Proteinase K (final 1.25 mg/mL, catalog no. 11-403B) and 10% SDS (catalog no. 97062-964) for 1 hour at 60°C and allowed to cool to room temperature. An equal volume of phenol:chloroform:isoamyl alcohol (25:24:1, vol/vol, catalog no. 97062-238) was added, inverted several times, and spun at 12,000 × *g* for 5 minutes at room temperature. The supernatant was transferred to a fresh tube, and another round of PCI alcohol extraction was performed, followed by a round of chloroform extraction. Finally, the supernatant was treated with 0.1 volume of 3 M NaOAc (pH 7.5) and 2.5 volumes of ice-cold 100% ethanol. The solution was mixed well by inversion and incubated at −20°C overnight. The DNA solution was centrifuged at 13,000 × *g* for 20 minutes at room temperature, and the pellet was washed twice with 70% ethanol (centrifuged at 13,000 × *g*, 2 minutes, room temperature). Ethanol was removed by air drying for 15–30 minutes, and nuclease-free water was used to resuspend the final DNA pellet.

#### DNA clean-up and gel electrophoresis

Freshwater and seawater underwent DNA purification via Qiagen’s DNeasy PowerClean Pro Cleanup Kit (catalog no. 12997-50) according to the manufacturer’s instructions to remove both color and PCR inhibitors. To visualize DNA for all experiments, agarose gel was prepared in TAE buffer and SYBR green (catalog no. S7563, Invitrogen) and ran for 50 minutes at 90 V with the Generuler Plus DNA ladder (catalog no. SM1331, Thermo Fisher Scientific). To assess the quality of the DNA, 2 µL of DNA was used for the Nanodrop (catalog no. ND-1000, Thermo Fisher Scientific).

#### Sequencing

After DNA extraction from metagenomic sources and prior to DNA library construction, freshwater (accession no. PRJNA849620), seawater (accession no. PRJNA849620), and wastewater (accession no. PRJNA849620) samples were sequenced via shotgun metagenomic sequencing (Novogene, CA, USA). Briefly, DNA was randomly sheared into shorter fragments, end-repaired, A-tailed, and further ligated with Illumina adapters using the NEBNext Ultra II library prep kit following the manufacturer’s instructions. Resulting fragments were size-selected and quantified through Qubit and qPCR, and size distribution was detected with a fragment analyzer. Libraries were pooled and loaded onto a NovaSeq6000 SP Flowcell for sequencing to generate paired-end reads. Fragments were sequenced to yield ~1 M PE150 reads per sample. Raw data were examined with FastQC (version 0.11.5), trimmed with Trimmomatic (version 0.36) using default options, and deduplicated with BBtools (version 38.22). Quality of the post-trimmed reads was examined with FastQC (version 0.11.5). Reads were assembled into contigs via MEGAHIT (version 1.2.9) with the following configurations: meta-sensitive, minimum contig length 300 ≤ 2,000 (35). Finally, a 0.04% subset of the wastewater crude extract reads were randomly extracted via Geneious (version 2023.0.4) and mapped to the contig WW54451 (Table S3).

### DNA library construction via E-LASL

E-LASL with slight modifications was used to construct expressible genomic (ФT4), multigenomic (ФHP3, ФES17, ФHC8A, Ф6947, and Ф6948), and metagenomic (freshwater, seawater, and wastewater) libraries. Briefly, 500 ng of DNA was digested with 0.2 units of MlucI (catalog no. R0538S, New England Biolabs) for either 5 or 10 minutes at 37°C, and the enzyme was inactivated via PCI (25:24:1) purification that consisted of two rounds of PCI, one round of chloroform, DNA precipitation in 95% ethanol, DNA wash with 70% ethanol, an overnight dry, and resuspension in 6 µL of nuclease-free water. Then, the EcoR I/Xho I linker (AATTCGGCTCGAG, catalog no. 26-3100-04, GeneLink) was ligated overnight at 16°C with ΦT4 ligase. The ligated products were amplified with PCR SuperMix (catalog no. 10572014) using linker-targeted primers (CCATGACTCGAGCCGAATT, Integrated DNA Technologies) following the manufacturer’s instructions. Amplified PCR products were cloned into pBAD via TOPO-TA cloning (catalog no. K430040, Thermo Fisher Scientific) and incubated at room temperature for 30 minutes. Cloned plasmids were transformed into TOP10 cells via chemical transformation. Then, all transformed cells were plated on LB plates with 0.2% glucose and 100 µg/mL ampicillin, and all colonies were harvested and stored at −80°C. Before harvest, 10 random colonies were picked for confirmation. They were grown overnight in ampicillin, had their plasmids extracted and their inserts amplified via PCR and analyzed in agarose gel with the conditions mentioned above (13).

### Confirmation and validation of the DNA libraries via agarose electrophoresis and sequencing

For each DNA library, 10 µL of frozen stock was used to inoculate each overnight culture for a total of five cultures per library. Each culture had a total of 2 mL of LB and incubated overnight at 37°C. Plasmids were extracted with the Qiagen’s Miniprep Kit, and 10 µL of plasmids was used for PCR amplification using the library primers. These inserts were analyzed with gel electrophoresis, and next-generation sequencing was performed as described above (Novogene, CA, USA). To estimate the rate of contamination, raw reads from all DNA libraries were assessed via ViromeQC (version 1.0, -w environmental for metagenomic reads) (15).

Raw reads were trimmed with BBTools (version 38.22) using default settings with the addition of duplicate reads and optical duplicates removal. Clean reads were assembled into contigs with MEGAHIT (version 1.2.9) using the meta-sensitive default settings. Removal of redundant contigs was performed using CD-HIT-EST (version 4.8.1) with at least 95% identities (35, 36). Genes were called and annotated with first Prokka (version 1.14.5) and then RAST (version 1.073) to have the proportion of unknown and annotated genes (37, 38). Additionally, protein sequences were classified based on the KEGG system through GhostKoala (version 2.0, database of prokaryotes + viruses) as well as Pharokka (database version 1.2.0) with Phanotate as the gene predictor, meta-mode for metavirome samples, and mmseqs2 PHROGs database with default settings (17, 39). Coverage plots were made by mapping contigs to their reference genomes or assembled metagenomic contigs via Geneious (version 2023.0.4, medium sensitivity). To understand the differences between each biological replicate and library, assembled contigs from all biological replicates and all libraries were clustered via CD-HIT-EST with at least 95% identities.

To understand the gene length of the various phages in both genomic and multigenomic libraries, all previously called genes from ФT4 (accession no. AF158101.6), ФHP3 (accession no. KY608967), ФES17 (accession no. MN508615.2), ФHC8A (accession no. PQ280049), Ф6947 (accession no. ON637251), and Ф6948 (accession no. OL362272) were graphed based on their size (Table S6). Additionally, 15 viral contigs with more than 70% completion from each biome (freshwater, seawater, and wastewater) were extracted from the IMG/VR (version 4) database and their genes were graphed based on the size in basepair (40). To determine the viral portion, non-redundant contigs from all libraries were classified via DeepVirFinder (version 1.0), and all viral contigs were defined as the ones with the *P*-value < 0.05 (16). Non-viral contigs from both the genomic and multigenomic libraries were mapped via the Geneious mapper (version 2023.0.4, high sensitivity) to their respective phages, the vector pBAD, and finally, the host *E. coli* MG1655 (accession no. U00096.3).

### Data representation and statistical analysis

The number of clones needed for each library (genomic and multigenomic) was calculated based on the formula developed by Clarke and Carbon (13):


$$N=\frac{\ln\left(1-P\right)}{\ln\left(1-f\right)}$$

where *N* is the number of recombinants (clones), *P* is the desired probability that the DNA fragment will be cloned at least once in the library, and *f* is the proportion of the genome in a clone, which can be represented as the insert size over the total genome size (13). Since we do not have one defined insert size, we used the average size estimated from the agarose gel.

Each DNA library was sampled, cultured, and sequenced independently at least five times. For statistical significance, one-way ANOVA was performed on the graphs showing the total number of genes per library, total number of contigs per library, and unique clusters between replicates and libraries. Two-way ANOVA was performed on the graph showing the total number of hypothetical vs annotated proteins per library. Kruskal-Wallis test was performed on the coverage of metagenomic DNA libraries graph. *P < 0.05; **P < 0.01; ***P < 0.001; and ****P < 0.0001. As for the genome coverage per library, graphs show the percentage of the reference genome (genomic and multigenomic libraries) or contig (metagenomic libraries).

The proportion of cloned DNA libraries over their known or sequenced total nucleotides was calculated as follows:


$$Proportion\;\left(\%\right)=\frac{DNA\;library}{Reference\;genome}x\;100$$

where the “DNA library” is the total number of nucleotides (bp) sequenced from the cloned DNA libraries, and “Reference genome” refers to either the total number of nucleotides (bp) of the T4 phage for the genomic library, the combined total for the multigenomic library, or the sequenced total nucleotides from crude extracts for the metagenomic libraries.

Finally, libraries were denoted throughout as “G” for genomic, “MG” for multigenomic, “FW” for freshwater, “SW” for seawater, and “WW” for wastewater. Each independent biological sampling of the DNA libraries is denoted as “A,” “B,” “C,” “D,” and “E” for a total of five replicates.

## SUPPLEMENTAL MATERIAL

Supplemental material aem.01171-24-s0001.docx Fig. S1 to S7 and Tables S1 to S6.

ASM does not own the copyrights to Supplemental Material that may be linked to, or accessed through, an article. The authors have granted ASM a non-exclusive, world-wide license to publish the Supplemental Material files. Please contact the corresponding author directly for reuse.
