# Supplementary material for: Construction and characterization of DNA libraries from cultured phages and environmental viromes
Source: Appl Environ Microbiol. 2024 Sep 24;90(10):e01171-24. doi: 10.1128/aem.01171-24 (PMC11497775; doi:10.1128/aem.01171-24)
Supplement: Supplemental material — Fig. S1 to S7 and Tables S1 to S6. [file aem.01171-24-s0001.docx]

**SUPPLEMENTAL MATERIALS**

**CONSTRUCTION AND CHARACTERIZATION OF DNA LIBRARIES FROM CULTURED PHAGES AND ENVIRONMENTAL VIROMES**

**Carmen Gu Liu^12^, Brianna E. Thompson^3^, James D. Chang^1^, Lorna Min^4^, Anthony W. Maresso^12^**

1 Department of Molecular Virology and Microbiology, Baylor College of Medicine, Houston, Texas, 77030, U.S.A.

2 TAILΦR: Tailored Antibacterials and Innovative Laboratories for phage (Φ) Research, Baylor College of Medicine, Houston, Texas, 77030, U.S.A.

3 Department of BioSciences, Rice University, Houston, Texas, 77005, U.S.A.

4 Department of Medicine, Baylor College of Medicine, Houston, Texas, 77030, U.S.A.

**Correspondence**: Anthony W. Maresso (maresso@bcm.edu)


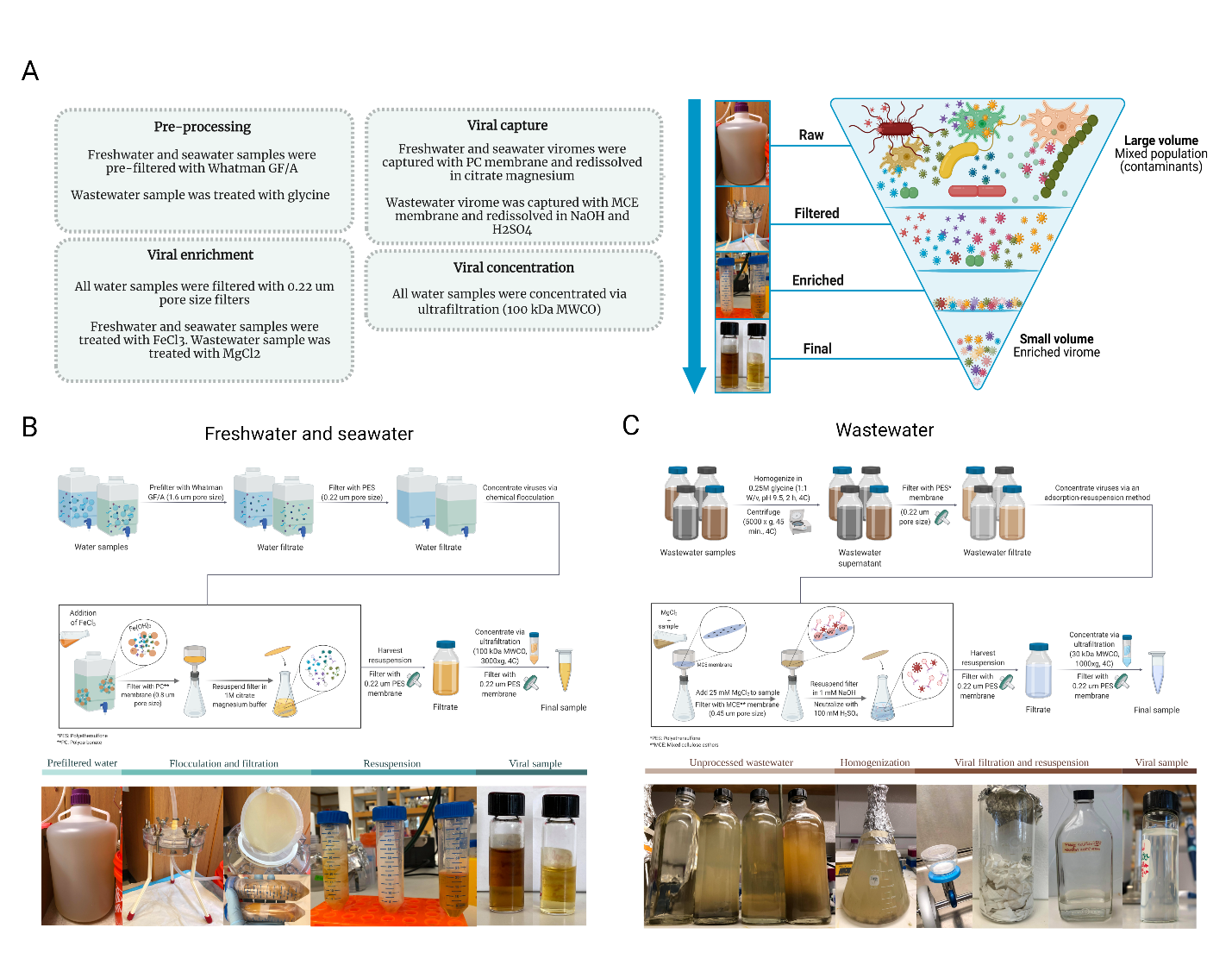


**Supplemental Figure 1. Processing environmental samples for viral enrichment, related to Figure 1.** A) To recover the viral fraction from water samples, different processing routes were employed. In summary, all water samples went through a pre-processing stage, viral enrichment, viral capture, and lastly, ultrafiltration. B) For both freshwater and seawater, viruses were captured with chemical flocculation via iron chloride, and were resuspended with citrate buffer. C) For the wastewater samples, viruses were captured with filtration via mixed cellulose esters (MCE) and were resuspended with NaOH and H_2_SO_4_.


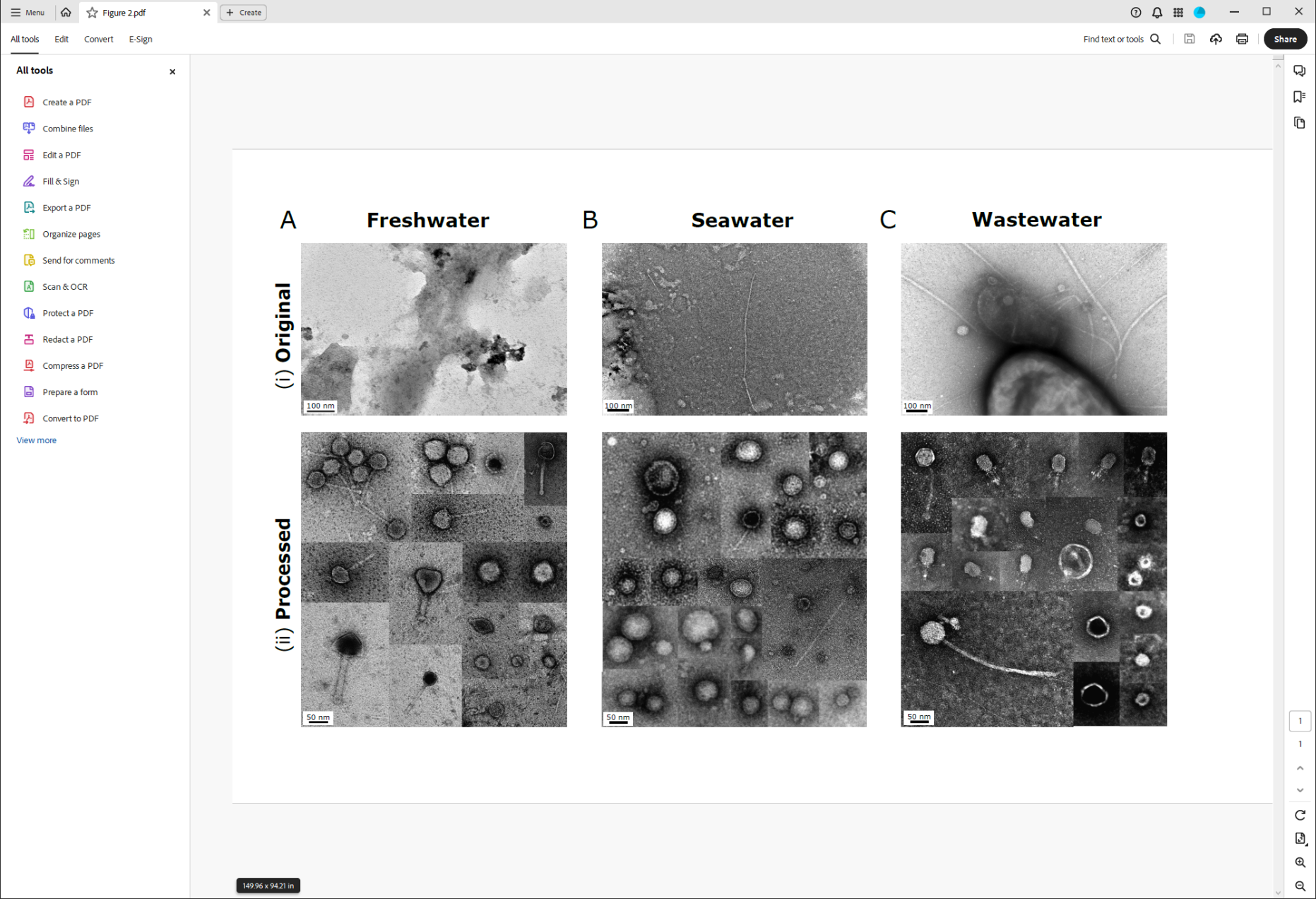


**Supplemental Figure 2. Detection of viral like particles via transmission electron microscopy (TEM).** To detect viral like particles from various environmental samples, 5 uL of both raw and final samples for A) freshwater, B) seawater, and C) wastewater were negatively stained and imaged with electron microscopy.


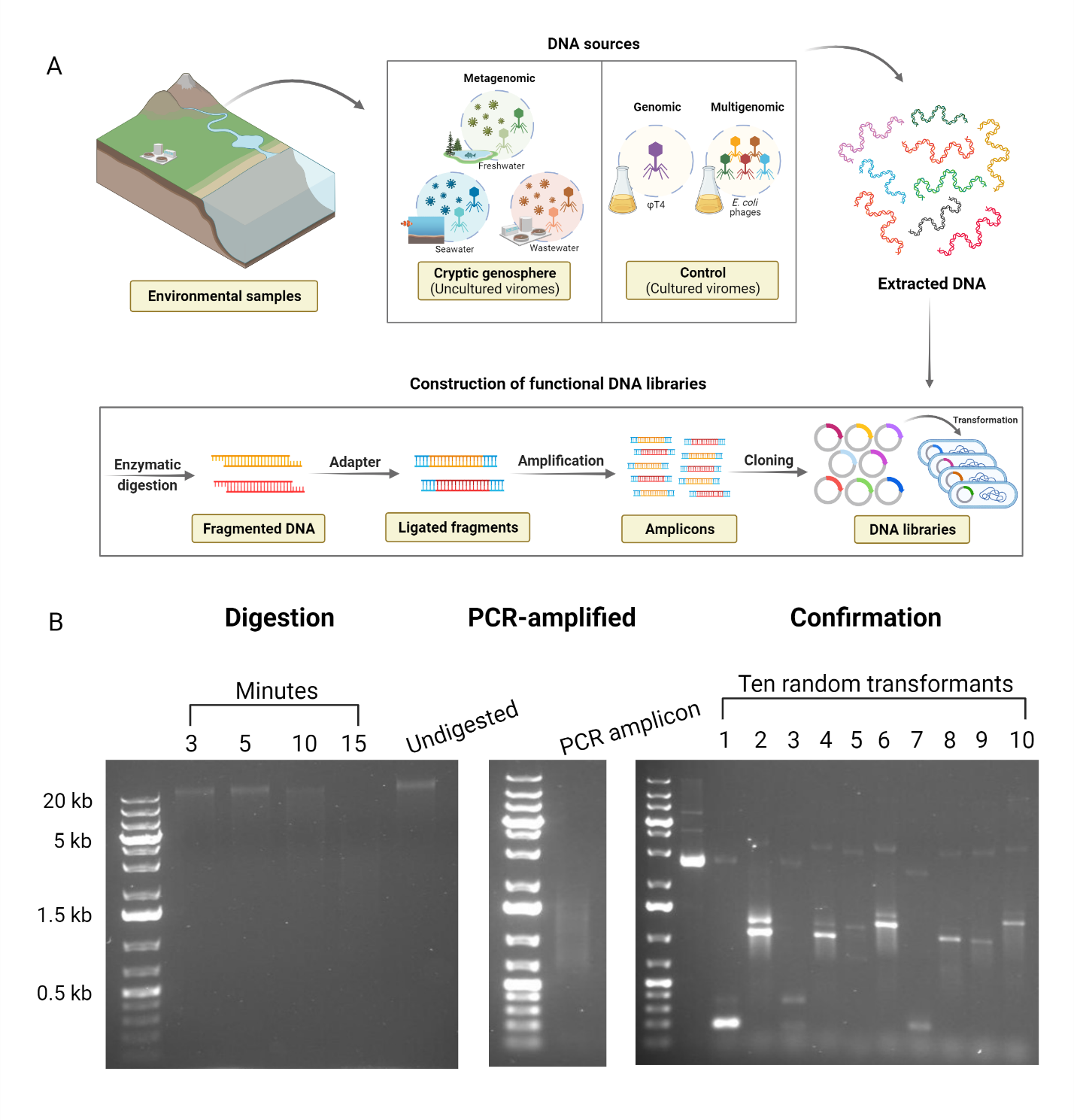


**Supplemental Fig****ure 3. The construction of DNA libraries from genomic, multigenomic, and metagenomic viral sources, related to Figure 1.** A) Schematic diagram of the generation of DNA libraries from various sources of DNA via the E-LASL (expressible linker-amplified shotgun library) approach with some slight modifications. These libraries consist of genomic (ΦT4), multigenomic (ФHP3, ФES17, ФHC8A, Ф6947, and Ф6948), metagenomic freshwater, seawater, and wastewater samples. Once the DNA was extracted, they were ligated to adapters, amplified via PCR leading to overhangs, cloned into plasmids, transformed, and stored at -80°C. B) DNA library construction gels showing multiple steps along the process. The first gel shows the digestion step with the MluCI enzyme: DNA ladder (lane 1), 3 minutes digestion (lane 2), 5 minutes digestion (lane 3), 10 minutes digestion (lane 4), 15 minutes digestion (lane 5), and undigested DNA (lane 6). The second gel shows the PCR-amplification step with the Taq polymerase: DNA ladder (lane 1) and PCR amplicon (lane 2). The third gel shows the confirmation step by isolating ten random transformants and amplifying the inserts: DNA ladder (lane 1), plasmid of transformant 1 (lane 2), PCR amplicon of ten random transformants (lanes 3-12). Agarose electrophoresis was performed for all libraries; only the representative gels are presented here.


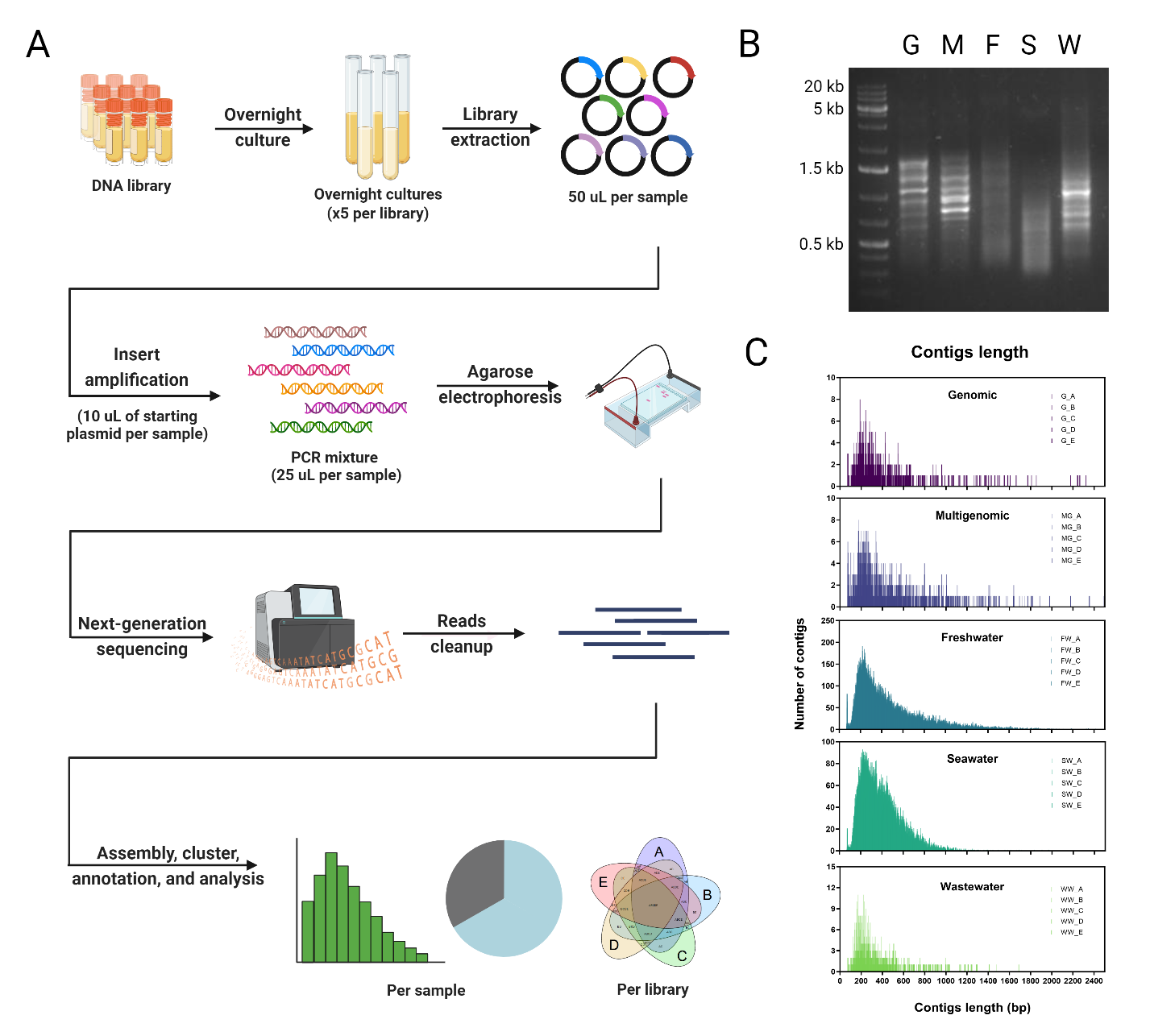


**Supplemental Figure 4. Confirmation and validation of the DNA libraries, related to Figures 1-5.** A) Schematic diagram for the steps taken to confirm DNA libraries via amplicons. 10 μL of frozen stock per library was inoculated into 2 mL of LB and incubated overnight at 37°C. Plasmids were extracted from each library with Qiagen’s Miniprep Kit, and inserts were amplified with primers that recognize the adaptors. These inserts were analyzed with gel electrophoresis and next-generation sequencing. B) Agarose electrophoresis analysis of the DNA library amplicons: DNA ladder (lane 1), Genomic library (lane 2), Multigenomic library (lane 3), Metagenomic Freshwater library (lane 4), Metagenomic Seawater library (lane 5), and Metagenomic Wastewater library (lane 6). C) Contigs length per replicate assembled from sequencing the libraries. Each independent biological sampling of the DNA libraries is denoted as “A”, “B”, “C”, “D”, and “E” for a total of five replicates.


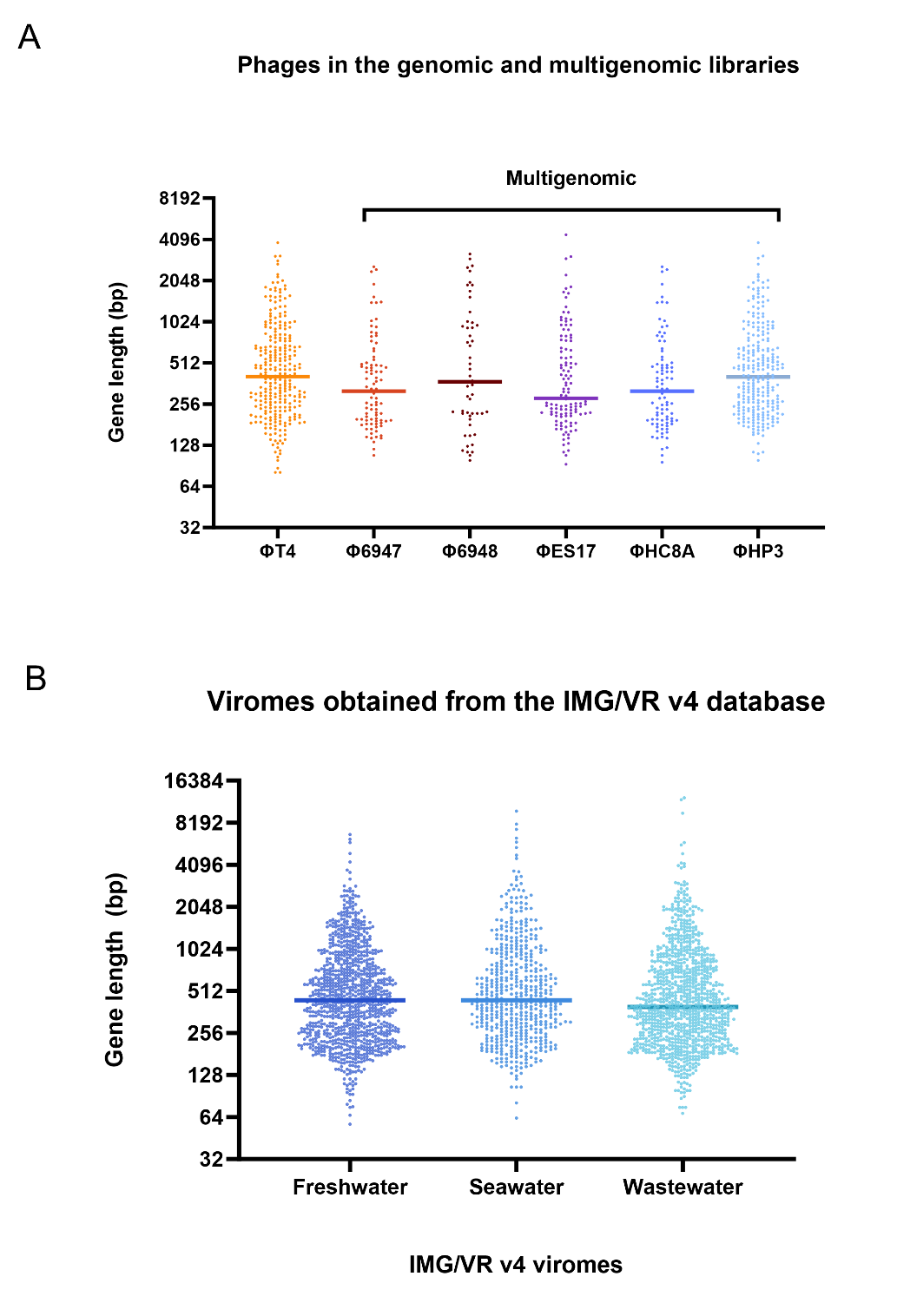


**Supplemental Figure 5. Size of the genes from all phages of the control libraries as well as environmental viromes from IMG/VR (v. 4).** A) All genes from phages of the genomic and multigenomic libraries were graphed based on their size (bp). B) Viral contigs with more than 70% completeness were randomly sampled from the IMG/VR v4 database and their genes were graphed based on their size (bp).


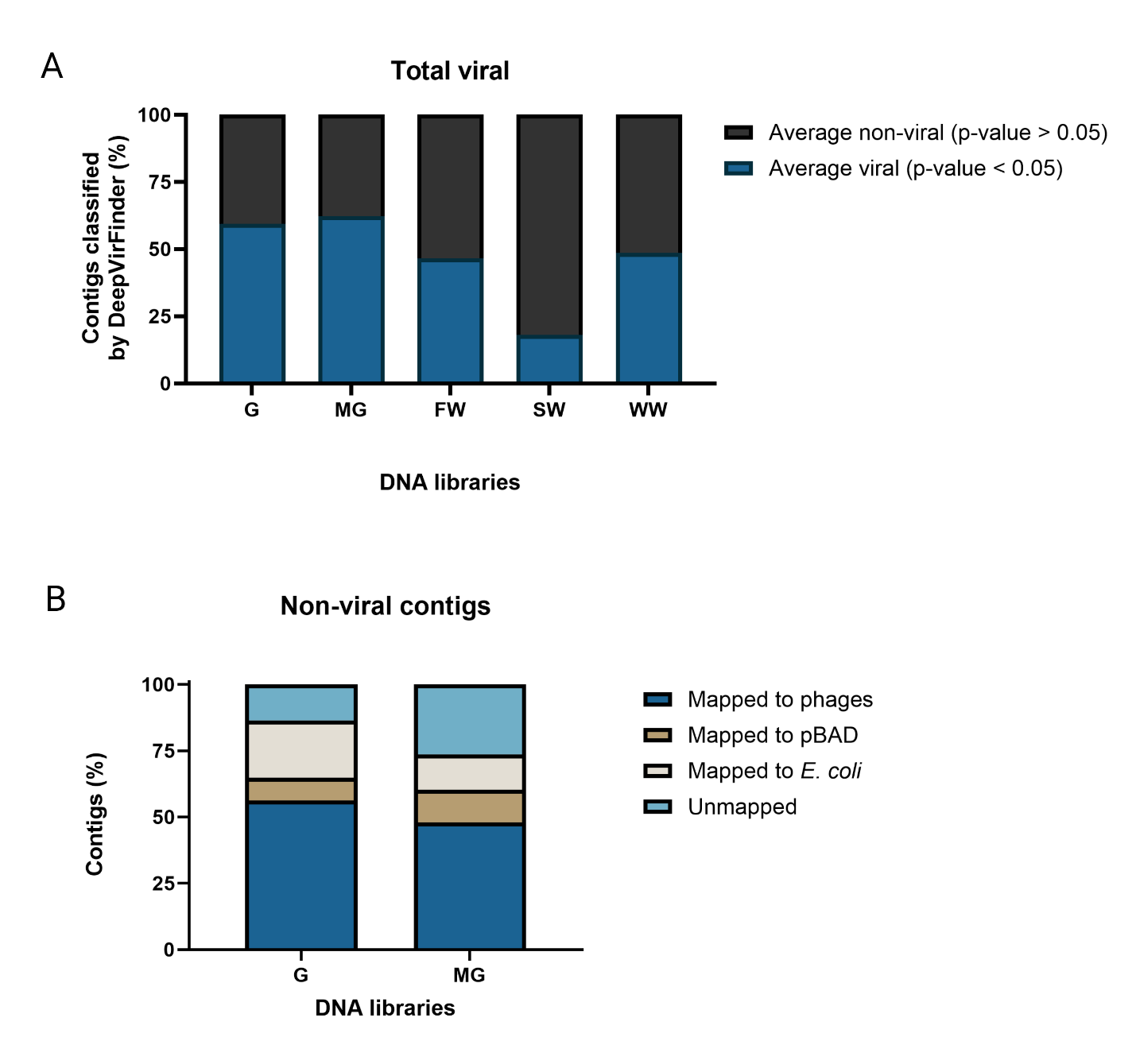


**Supplemental Figure 6. Proportion of viral vs non-viral contigs from all libraries.** Contigs from all libraries were classified with DeepVirFinder (v. 1.0) into A) viral vs non-viral contigs, and the non-viral portion from the genomic and multigenomic libraries were mapped via the Geneious mapper to their respective phages, the vector pBAD, and lastly, the *E. coli* MG1655 host.


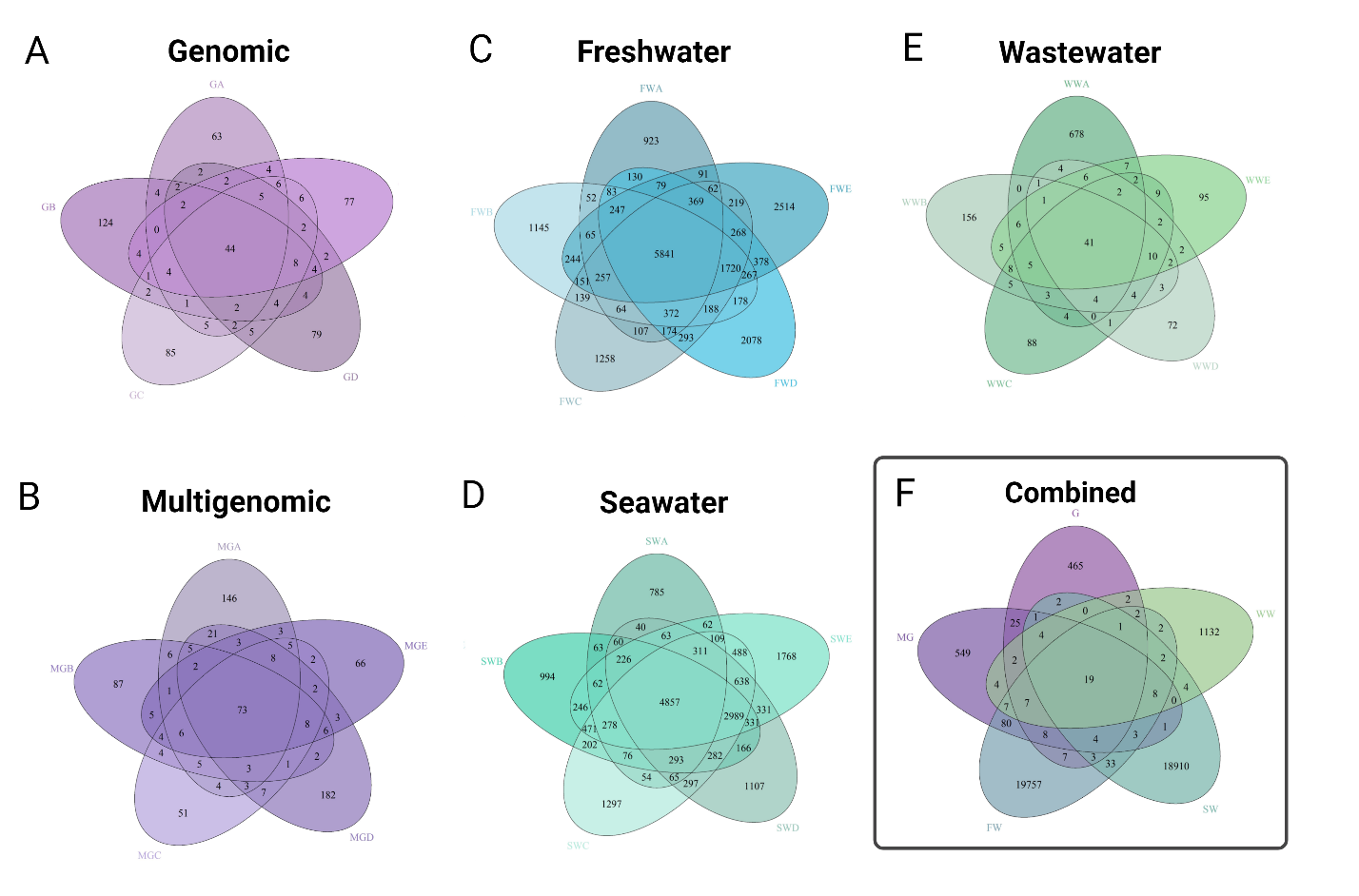


**Supplemental Figure 7. Venn diagrams show unique and shared DNA clusters in all DNA libraries, related to Figure 5.** DNA sequences from each library were clustered between replicates and represented as Venn diagrams, showing the exact counts of unique and shared DNA clusters between replicates: A) Genomic library, B) Multigenomic library, C) Freshwater library, D) Seawater library, and E) Wastewater library. In addition, sequences from all libraries were clustered and represented as a F) Venn diagram showing exact counts of unique and shared DNA clusters between libraries

**Supplemental Table 1. Volume and sources of collected freshwater, seawater, and wastewater for viral metagenomics**

| **Samples** | **Source type** | **Volume processed** | **Source City** |
| --- | --- | --- | --- |
| **Freshwater** | Creek | 57 L | Austin, TX |
|  | River | 57 L | Austin, TX |
|  | Lake | 57 L | Houston, TX |
|  | Wetland | 57 L | Houston, TX |
| **Seawater** | Gulf | 57 L | Galveston, TX |
|  | Bay | 57 L | La Porte, TX |
| **Wastewater** | Anaerobic digestion and influent | 9.5 L | Austin, TX  (Multiple wastewater treatment plants) |
|  | Influent | 6.6 L | Houston, TX  (Multiple wastewater treatment plants) |

**Supplemental Table 2. Shotgun metagenomics reads from the crude extracts of several water sources**

| **Samples** | **Freshwater virome** | **Seawater virome** | **Wastewater virome** |
| --- | --- | --- | --- |
| **Raw reads** | | | |
| Number of reads | 42,630,036 | 38,379,834 | 38,904,796 |
| Total number of bases (bp) | 6,394,505,400 | 5,756,975,100 | 5,835,719,400 |
| Number of duplicate reads | 349,139 (0.82%) | 409,227 (1.07%) | 32,439,443 (83.38%) |
| Quality Score Mean | 36.30 | 36.48 | 35.96 |
| GC percentage | 50.9% | 46.12% | 34.25% |
| **Post-trimming** | | | |
| Number of reads | 41,853,070 | 37,811,180 | 38,010,788 |
| Total number of bases (bp) | 6,212,745,821 | 5,588,919,859 | 5,625,341,690 |
| Number of duplicate reads | 347,804 (0.83%) | 401,340 (1.06%) | 32,050,475 (84.32%) |
| GC percentage | 50.9% | 46.11% | 34.25% |
| **Post-assembly** | | | |
| Number of contigs | 28,584 | 18,065 | 1,932 |
| Total length (bp) | 124,817,309 | 136,221,491 | 9,378,825 |
| N50 | 4574 | 15287 | 5305 |
| GC percentage | 50.29% | 45.81% | 46.17% |

**Supplemental Table 3. Highly prevalent *Bacillus* phage recovered from the wastewater metagenomic crude extract**

|  | **Number of reads** | **Percentage** | **Alignment result** |
| --- | --- | --- | --- |
| **Post-trimming Total** | 38,010,788 | 100% |  |
| **Post-trimming duplicated reads** | 32,050,475 | 84% of post-trimming total |  |
| **Randomized subset of 0.04%** | 15,204 | 0.04% of post-trimming total |  |
| **Mapping a randomized subset to WW54451 contig** | 13,055 | 0.034% of post-trimming total  85% of the randomized subset (0.04%) |  |
| **WW54451 contig** |  |  | - Bacillus phage Nf (29.4% identities) - Bacillus phage vB_BsuP-Goe1 (29.5% identities) |

**Supplemental Table 4. ViromeQC assessment of cloned DNA libraries**

| **Samples** | **SSU rRNA alignment rate (%)** | **LSU rRNA alignment rate (%)** | **Bacterial markers alignment rate (%)** |
| --- | --- | --- | --- |
| **Genomic** | | | |
| **G_A** | 0.21774 | 1.78440 | 0.00011 |
| **G_B** | 0.25877 | 2.14595 | 0.00010 |
| **G_C** | 0.49293 | 4.09119 | 0.00039 |
| **G_D** | 0.43682 | 3.63793 | 0.00024 |
| **G_E** | 0.37261 | 3.13905 | 0.00018 |
| **G average** | **0.35577** | **2.95970** | **0.00021** |
| **Multigenomic** | | | |
| **MG_A** | 0.57135 | 4.65310 | 0.00121 |
| **MG_B** | 0.18868 | 1.54205 | 0.00013 |
| **MG_C** | 0.29246 | 2.38745 | 0.00052 |
| **MG_D** | 0.32696 | 2.73686 | 0.00079 |
| **MG_E** | 0.42225 | 3.47336 | 0.00031 |
| **MG average** | **0.36034** | **2.95856** | **0.00059** |
| **Freshwater** | | | |
| **FW_A** | 0.39013 | 2.16641 | 0.25053 |
| **FW_B** | 1.38766 | 11.24792 | 0.10372 |
| **FW_C** | 0.98662 | 7.96813 | 0.14929 |
| **FW_D** | 1.92291 | 15.86241 | 0.08551 |
| **FW_E** | 1.92291 | 15.86241 | 0.08551 |
| **FW average** | **1.32205** | **10.62145** | **0.13491** |
| **Seawater** | | | |
| **SW_A** | 0.83723 | 4.23617 | 0.48339 |
| **SW_B** | 1.34138 | 10.12416 | 0.33226 |
| **SW_C** | 1.85506 | 14.57431 | 0.28654 |
| **SW_D** | 1.74332 | 13.50315 | 0.28362 |
| **SW_E** | 1.33610 | 10.52834 | 0.32633 |
| **SW average** | **1.42262** | **10.59323** | **0.34243** |
| **Wastewater** | | | |
| **WW_A** | 0.32491 | 1.72945 | 0.00081 |
| **WW_B** | 0.37614 | 3.03041 | 0.00078 |
| **WW_C** | 0.37800 | 3.07587 | 0.00017 |
| **WW_D** | 0.26488 | 2.11016 | 0.00018 |
| **WW_E** | 0.36120 | 2.96224 | 0.00019 |
| **WW average** | **0.34103** | **2.58163** | **0.00043** |

**Supplemental Table 5. Presence and coverage of phage toxic products in the genomic and multigenomic DNA libraries**

| **DNA library** | **Phage** | **Phage products present** | **Known to cause toxicity in *E. coli host*** | **Covered by DNA library** |
| --- | --- | --- | --- | --- |
| **Genomic** | T4 | Endolysin (gene *e*) | Yes, with the help of holin (gene *t*)^1^ | Yes |
| **Genomic** | T4 | Holin (gene *t*) | Yes, with the help of endolysin (gene *e*)^1^ | Yes |
| **Genomic** | T4 | Nucleoid disruption protein (*ndd*) | Yes ^1,2^ | Yes |
| **Genomic** | T4 | Endoribonuclease (*regB*) | Yes ^1,3^ | Yes |
| **Multigenomic** | HP3 | Holin | Not tested | Yes |
| **Multigenomic** | HP3 | Lysozyme | Not tested | Yes |
| **Multigenomic** | HC8A | Lysozyme | Not tested | No |
| **Multigenomic** | HC8A | Lysin | Not tested | No |
| **Multigenomic** | ES17 | Lysin | Not tested | Yes |
| **Multigenomic** | 6948 | Holin | Not tested | Yes |
| **Multigenomic** | 6947 | Lysin | Not tested | No |
| **Multigenomic** | 6947 | Lysozyme | Not tested | No |

**Supplemental Table 6. Bacterial strains, phages, and plasmids used in this study as well as their characteristics and/or genotype and references**

| **Bacterial strain, phage, and plasmid** | **Characteristics and/or genotype** | **Reference, source, and identifier** |
| --- | --- | --- |
| **Bacteria** | | |
| *Escherichia coli* K12 (BW25113/ME9062) | rrnB DElacZ4787 HsdR514 DE(araBAD)567 DE(rhaBAD)568 rph-1 | KEIO collection ^4,5^ |
| **Phages** | | |
| ФT4 | *Escherichia coli* phage, ATCC strain 11303-B4 | ATCC (Catalog #11303-B4), accession No. AF158101.6 |
| ФHP3 | *Escherichia coli* phage | Our lab ^6^, accession No. KY608967 |
| ФES17 | *Escherichia coli* phage | TAILФR LABS ^7^, accession No. MN508615.2 |
| ФHC8A | *Escherichia coli* phage | TAILФR LABS ^7^, accession No. PQ280049 |
| Ф6947 | *Escherichia coli* phage | TAILФR LABS ^7^, accession No. ON637251 |
| Ф6948 | *Escherichia coli* phage | TAILФR LABS ^7^, accession No. OL362272 |
| Freshwater virome | Environmental samples collected from lake, river, creek and wetland around Austin and Houston, TX | Accession No. SAMN29097100 |
| Seawater virome | Environmental samples collected from beaches around Houston, TX | Accession No. SAMN29101438 |
| Wastewater virome | Wastewater samples collected from treatment plants around Austin and Houston, TX | Accession No. SAMN29102399 |
| Genomic DNA library | DNA library made from ФT4 ATCC strain 11303-B4, replicate | Accession No. PRJNA1120465  Replicate A: SAMN41694220  Replicate B: SAMN41694221  Replicate C: SAMN41694222  Replicate D: SAMN41694223  Replicate E: SAMN41694224 |
| Multigenomic DNA library | DNA library made from ФHP3, ФES17, ФHC8A, Ф6947, and Ф6948 | Accession No. PRJNA1120465  Replicate A: SAMN41694225  Replicate B: SAMN41694226  Replicate C: SAMN41694227  Replicate D: SAMN41694228  Replicate E: SAMN41694229 |
| Freshwater DNA library | DNA library made from the freshwater virome | Accession No. PRJNA1120465  Replicate A: SAMN41694521  Replicate B: SAMN41694522  Replicate C: SAMN41694523  Replicate D: SAMN41694524  Replicate E: SAMN41694525 |
| Seawater DNA library | DNA library made from the seawater virome | Accession No. PRJNA1120465  Replicate A: SAMN41694526  Replicate B: SAMN41694527  Replicate C: SAMN41694528  Replicate D: SAMN41694529  Replicate E: SAMN41694530 |
| Wastewater DNA library | DNA library made from the wastewater virome | Accession No. PRJNA1120465  Replicate A: SAMN41694531  Replicate B: SAMN41694532  Replicate C: SAMN41694533  Replicate D: SAMN41694534  Replicate E: SAMN41694535 |
| **Plasmid** | | |
| pBAD | Cloning plasmid | Thermo Fisher Scientific (Catalog #K430040) |

**References**

1 Mohanraj, U., Wan, X., Spruit, C. M., Skurnik, M. & Pajunen, M. I. A Toxicity Screening Approach to Identify Bacteriophage-Encoded Anti-Microbial Proteins. *Viruses* **11**, doi:10.3390/v11111057 (2019).

2 Bouet, J. Y., Campo, N. J., Krisch, H. M. & Louarn, J. M. The effects on Escherichia coli of expression of the cloned bacteriophage T4 nucleoid disruption (ndd) gene. *Mol Microbiol* **20**, 519-528, doi:10.1046/j.1365-2958.1996.5411067.x (1996).

3 Saida, F., Uzan, M. & Bontems, F. The phage T4 restriction endoribonuclease RegB: a cyclizing enzyme that requires two histidines to be fully active. *Nucleic Acids Res* **31**, 2751-2758, doi:10.1093/nar/gkg377 (2003).

4 Baba, T. *et al.* in *Molecular Systems Biology* Vol. 2 (2006).

5 Yamamoto, N. *et al.* in *Molecular Systems Biology* Vol. 5 (2009).

6 Green, S. I. *et al.* Bacteriophages from ExPEC Reservoirs Kill Pandemic Multidrug-Resistant Strains of Clonal Group ST131 in Animal Models of Bacteremia. *Sci Rep* **7**, 46151, doi:10.1038/srep46151 (2017).

7 Terwilliger, A. L. *et al.* Tailored Antibacterials and Innovative Laboratories for Phage (Phi) Research: Personalized Infectious Disease Medicine for the Most Vulnerable At-Risk Patients. *Phage (New Rochelle)* **1**, 66-74, doi:10.1089/phage.2020.0007 (2020).
